# Subcritical Water Hydrolysis of Peptides: Amino Acid Side-Chain Modifications

**DOI:** 10.1007/s13361-017-1676-1

**Published:** 2017-05-17

**Authors:** Thomas Powell, Steve Bowra, Helen J. Cooper

**Affiliations:** 1 School of Biosciences, University of Birmingham, Edgbaston, Birmingham, B15 2TT UK; 2 Phytatec (UK) Ltd., Plas Gogerddan, Aberystwyth, SY23 3EB UK

**Keywords:** Subcritical water, Amino acid side chain modification, Peptide, Cysteine, Methionine, Tryptophan

## Abstract

Previously we have shown that subcritical water may be used as an alternative to enzymatic digestion in the proteolysis of proteins for bottom-up proteomics. Subcritical water hydrolysis of proteins was shown to result in protein sequence coverages greater than or equal to that obtained following digestion with trypsin; however, the percentage of peptide spectral matches for the samples treated with trypsin were consistently greater than for those treated with subcritical water. This observation suggests that in addition to cleavage of the peptide bond, subcritical water treatment results in other hydrolysis products, possibly due to modifications of amino acid side chains. Here, a model peptide comprising all common amino acid residues (VQSIKCADFLHYMENPTWGR) and two further model peptides (VCFQYMDRGDR and VQSIKADFLHYENPTWGR) were treated with subcritical water with the aim of probing any induced amino acid side-chain modifications. The hydrolysis products were analyzed by direct infusion electrospray tandem mass spectrometry, either collision-induced dissociation or electron transfer dissociation, and liquid chromatography collision-induced dissociation tandem mass spectrometry. The results show preferential oxidation of cysteine to sulfinic and sulfonic acid, and oxidation of methionine. In the absence of cysteine and methionine, oxidation of tryptophan was observed. In addition, water loss from aspartic acid and C-terminal amidation were observed in harsher subcritical water conditions.

**Electronic supplementary material:**

The online version of this article (doi:10.1007/s13361-017-1676-1) contains supplementary material, which is available to authorized users.

**Graphical Abstract:**
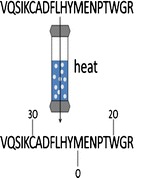
ᅟ

## Introduction

Subcritical water (SCW) exists at a temperature between 100 °C and 374 °C and a pressure less than 22 MPa (i.e., below its critical point). In the subcritical state, the ionic product of water, K_w_, increases with temperature, resulting in the formation of hydronium (H_3_O^+^) and hydroxide (OH^–^) ions, enabling SCW to act as either an acid or base catalyst [1]. We have previously investigated SCW as an alternative to tryptic digestion in the bottom-up analysis of proteins [2]. In that work we demonstrated that SCW hydrolysis of three standard proteins, hemoglobin, bovine serum albumin (BSA), and beta casein, followed by LC MS/MS of the hydrolysis products, resulted in protein sequence coverages comparable to, or greater than, that of trypsin. Furthermore, SCW was shown to be effective at maintaining post-translational modifications (PTMs) under certain conditions. Despite the high sequence coverages obtained through SCW hydrolysis, the percentage of peptide spectral matches (PSMs) was low in comparison to the corresponding tryptic digests. This observation suggests that in addition to hydrolysis of the peptide bond, SCW treatment results in other chemical reactions, potentially including modification of amino acid side chains.

In the present study, we sought to determine the effect of SCW on amino acid side chains using a model peptide approach. The synthetic peptide VQSIKCADFLHYMENPTWGR, which contains all 20 commonly-occurring amino acid residues, was synthesized and treated with SCW at one of four temperature points (140, 160, 180, 200 °C) for 10 min. The influence of SCW temperature on peptide modifications was evaluated at each temperature point by use of tandem mass spectrometry (either collision-induced dissociation or electron transfer dissociation). The results showed that the most common modification was oxidation of cysteine and methionine residues. Cysteine can exist in a number of oxidation states, most commonly the free thiol or the disulfide; however, oxidation to sulfenic (SOH), sulfinic (SO_2_H), and sulfonic (SO_3_H) acids is also known [3]. Our results show SCW mediated oxidation of cysteine to sulfinic and sulfonic acid. In addition, water loss and C-terminal amidation were observed under the harshest SCW conditions. The induction of these modifications was confirmed by SCW treatment of a second peptide, VCFQYMDRGDR. We also investigated the effect of SCW on peptides that do not contain cysteine or methionine. The results demonstrate that tryptophan is also susceptible to oxidation.

## Experimental

### Preparation of Samples for Mass Spectrometry

#### Samples

Three model peptides, VQSIKCADFLHYMENPTWGR, VCFQYMDRGDR, and VQSIKADFLHYENPTWGR, were synthesized by Genic Bio Ltd. (Shanghai, China) and used without further purification. The peptides were diluted to ~15 μM in water (J. T. Baker, Deventer, The Netherlands).

For some experiments (see text) cysteine residues were alkylated prior to SCW treatment. In those experiments, a 15 mL solution containing ~15 μM peptide, ~14 mM iodoacetamide (Sigma Aldrich, Gillingham, UK), and 100 mM ammonium bicarbonate was incubated for 20 min at room temperature in the dark. In experiments where a thiol was introduced as a quenching agent, ~14 mM DTT was added following alkylation, and incubated in the dark at room temperature for 30 min.

#### SCW Hydrolysis

15 mL of 15 μM of synthetic peptide was placed inside a reaction tube consisting of stainless steel metal piping, 200 mm × 16.4 mm, capped using SS tube fitting, reducing union, 3/4′′ × 1/4′′ (Swagelok, Manchester, UK). SCW hydrolysis was performed at temperatures of 140 °C, 160 °C, 180 °C, and 200 °C. Reaction tubes were placed in the oven preset to 30 °C and allowed to equilibrate for at least 10 min prior to increasing the temperature of the oven. The reaction temperature was monitored by use of a thermocouple attached to one of the reaction tubes. From this starting temperature it took 5 min 10 s to reach 140 °C, 6 min 40 s to reach 160 °C, 7 min to reach 180 °C, and 8 min to reach 200 °C. Reactions were held stably at these temperatures for the reaction times of 10 min. The selected residency (i.e., 10 min), began after reaching the chosen temperature. Hydrolysis was quenched by placing the reaction tubes into a bucket of ice for 5 min. One mL aliquots of the SCW products were then stored at –20 °C until analysis. Samples were diluted to a concentration of ~3 μM in 50:50 water: methanol (J.T. Baker, Deventer, The Netherlands), 0.1% formic acid (Fisher Scientific, Loughborough, UK), and introduced to the mass spectrometer by electrospray ionisation.

### Direct Infusion Electrospray Mass Spectrometry

All mass spectrometry experiments were performed on a Thermo Fisher Orbitrap Elite (Thermo Fisher, Bremen, Germany). Data acquisition was controlled by Xcalibur 2.1 (Thermo Fisher).

All direct infusion electrospray mass spectra and tandem mass spectra were recorded in the Orbitrap at a resolution of 240,000 at *m*/*z* 400.

#### CID

The automatic gain control (AGC) target was 5 × 10^4^ charges with maximum injection time of 300 ms. CID was performed in the ion trap using helium at normalized collision energy of 35% and the fragments were detected in the Orbitrap. Width of the precursor isolation window was 1.5 *m*/*z*.

#### ETD

The AGC target was 5 × 10^4^ charges with maximum injection time 300 ms. Supplemental activation (sa) ETD was performed in the ion trap with fluoranthene reagent ions (AGC target for reagent ions was 1 × 10^5^ charges with a maximum injection time of 100 ms) and a normalized collision energy (sa) of 25%. Width of the precursor isolation window was 1.5 *m*/*z*. Fragment ions were detected in the Orbitrap.

### LC-MS/MS

LC-MS/MS was performed in circumstances where the site of modification was ambiguous (see text).

#### Liquid Chromatography

Peptides were separated using online reversed phase LC (Dionex Ultimate 3000), using a binary solvent system consisting of mobile phase A [water (J.T. Baker, Deventer, The Netherlands)/0.1% formic acid (Fisher Scientific, Loughborough, UK)] and mobile phase B [acetonitrile (J.T. Baker)/0.1% formic acid (Fisher Scientific)]. Peptide hydrolysates were loaded onto a C18 column (LC Packings, Sunnyvale, CA, USA), in mobile phase A and separated over a linear gradient from 3.2% to 44% mobile phase B with a flow rate of 350 nL/min. The column was then washed with 90% mobile phase B before re-equilibrating at 3.2% mobile phase B. Samples eluted directly via a Triversa Nanomate nanoelectrospray source (Advion Biosciences, Ithaca, NY, USA) into the Orbitrap Elite mass spectrometer.

#### CID

The mass spectrometer performed a full MS scan (*m*/*z* 350–1800) and subsequent MS/MS scans of the seven most abundant ions that had a charge state >1. Survey scans were acquired in the Orbitrap with a resolution of 60,000 at *m*/*z* 400. The AGC target for the survey scans was 10^6^ charges with maximum injection time of 1 s. CID was performed in the ion trap using helium at normalized collision energy of 35%, and the fragments were detected in the Orbitrap (resolution 60,000 at *m*/*z* 400). Width of the precursor isolation window was 2 *m*/*z*. AGC target for CID was 5 × 10^4^ charges with a maximum injection time of 100 ms.

#### ETD

The mass spectrometer performed a full MS scan (*m*/*z* 350–1800) and subsequent MS/MS scans of the seven most abundant ions that had a charge state >1. Survey scans were acquired in the Orbitrap with a resolution of 60,000 at *m*/*z* 400. The AGC target for the survey scans was 10^6^ charges with maximum injection time of 1 s. ETD was performed in the linear ion trap with fluoranthene ions with a maximum fill time of 100 ms. The fragments were detected in the Orbitrap (resolution 60,000 at *m*/*z* 400). Width of the precursor isolation window was 2 *m*/*z*. Precursor ions were activated for 100 ms (charge-dependent activation time was enabled). The AGC target for ETD was 5 × 10^4^ charges. Supplemental activation was used with normalized collision energy of 25%.

## Results

In order to determine the effects of SCW hydrolysis on the side chains of amino acid residues, a model peptide that incorporates all 20 natural amino acids was designed and synthesized, VQSIKCADFLHYMENPTWGR. An arginine residue was placed at the C-terminus of the peptide in order to allow efficient generation of a ‘y’ or ‘z’ fragment ion series following fragmentation. The acidic glutamate and aspartate residues, which are known to direct backbone cleavage in SCW conditions [2], were separated by five amino acid residues. The direct infusion electrospray mass spectrum of the peptide is shown in Supplementary Figure 1, with peak assignments detailed in Supplementary Table 1. (Note, there are some low abundance peaks that correspond to impurities resulting from incorrect synthesis of the model peptide). Samples of the peptide were subjected to SCW hydrolysis at 140 °C for 10 min, 160 °C for 10 min, 180 °C for 10 min, and 200 °C for 10 min, and the resulting hydrolysates were analyzed by direct infusion electrospray mass spectrometry. A summary of the peaks observed is given in Table 1. Selected peaks were isolated and fragmented by both CID and ETD MS/MS, as described below.

**Table 1:** Ions Observed Following SCW Hydrolysis of VQSIKCADFLHYMENPTWGR at 140 °C, 160 °C, 180 °C, and 200 °C for 10 min

*m*/*z*	z	Calculated mass (Da)	Measured mass (Da)	Peptide	ΔPPM
140 °C 10 min					
517.5779	3	1549.7136	1549.7119	FLHYMENPTWGR	–1.1
522.9094	3	1565.7085	1565.7064	FLHYMENPTWGR (+O)	–1.4
617.0220	4	2464.0701	2464.0589	VQSIKCADFLHYMENPTWGR (+K+2O)	–4.5
621.0207	4	2480.0650	2480.0537	VQSIKCADFLHYMENPTWGR (+K+3O)	–4.5
809.7109	3	2426.1147	2426.1109	VQSIKCADFLHYMENPTWGR (+2O)	–1.6
815.0426	3	2442.1096	2442.1060	VQSIKCADFLHYMENPTWGR (+3O)	–1.5
820.3743	3	2458.1045	2458.1011	VQSIKCADFLHYMENPTWGR (+4O)	–1.4
1214.0621	2	2426.1147	2426.1096	VQSIKCADFLHYMENPTWGR (+2O)	–2.1
1222.0594	2	2442.1096	2442.1042	VQSIKCADFLHYMENPTWGR (+3O)	–2.2
160 °C 10 min					
522.9093	3	1565.7085	1565.7061	FLHYMENPTWGR (+O)	–1.6
530.2266	3	1587.6689	1587.6580	FLHYMENPTWGR (+ K)	–6.9
645.7868	2	1289.5611	1289.5590	HYMENPTWGR	–1.6
775.8629	2	1549.7136	1549.7112	FLHYMENPTWGR	–1.5
809.7106	3	2426.1147	2426.1100	VQSIKCADFLHYMENPTWGR (+2O)	–2.0
815.0424	3	2442.1096	2442.1054	VQSIKCADFLHYMENPTWGR (+3O)	–1.7
180 °C 10 min					
457.2785	1			Unassigned	
517.5801	3	1549.7136	1549.7185	FLHYMENPTWGR	3.1
522.9117	3	1565.7085	1565.7133	FLHYMENPTWGR (+O)	3.0
577.2600	2	1152.5022	1152.5054	YMENPTWGR	2.8
583.7703	2	1165.5226	1165.5260	FLHYMENPT (C-terminal amidation+O)	3.0
585.2570	2	1168.4971	1168.4994	YMENPTWGR (+O)	2.0
616.3218	1	615.3129	615.3145	PTWGR	2.6
645.7897	2	1289.5611	1289.5648	HYMENPTWGR	2.9
653.7871	2	1305.5560	1305.5596	HYMENPTWGR (+O)	2.8
727.3919	1			Unassigned	
775.8662	2	1549.7136	1549.7178	FLHYMENPTWGR	2.7
783.8634	2	1565.7085	1565.7122	FLHYMENPTWGR (+O)	2.4
867.8906	2	1733.7621	1733.7666	ADFLHYMENPTWGR (-H_2_O+O)	2.6
200 °C 10 min					
457.2801	1			Unassigned	
517.1089	1			Unassigned	
519.2702	1	518.2601	518.2629	TWGR	5.4
573.3761	1			Unassigned	
577.2625	2	1152.5022	1152.5104	YMENPTWGR	7.1
583.7728	2	1165.5226	1165.5310	FLHYMENPT (C-terminal amidation+O)	7.2
585.2600	2	1168.4971	1168.5054	YMENPTWGR (+O)	7.1
653.7898	2	1305.5560	1305.5650	HYMENPTWGR (+O)	6.9

**Figure 1:**
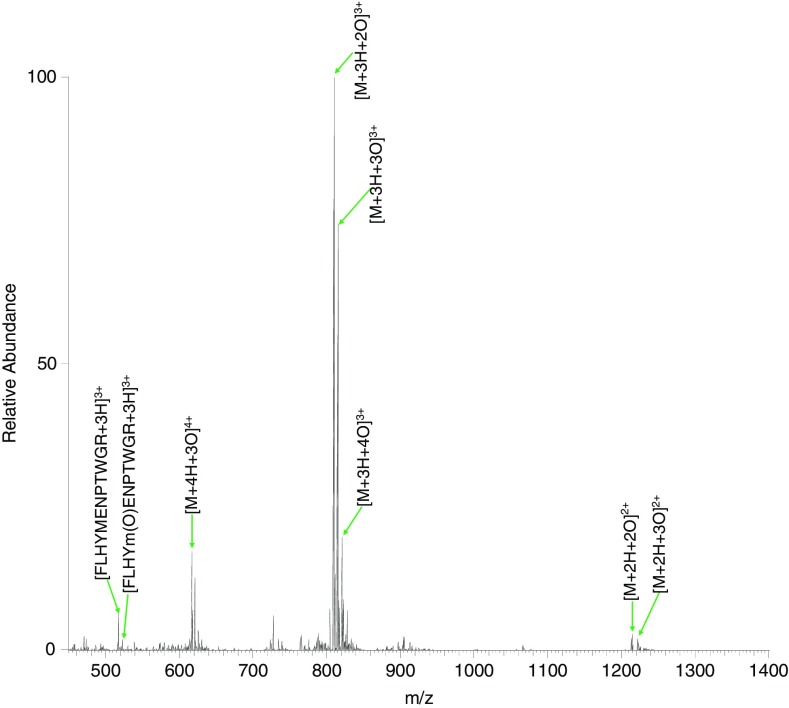
Direct infusion electrospray MS of peptide VQSIKCADFLHYMENPTWGR treated with SCW at 140 °C for 10 min

**Figure 2:**
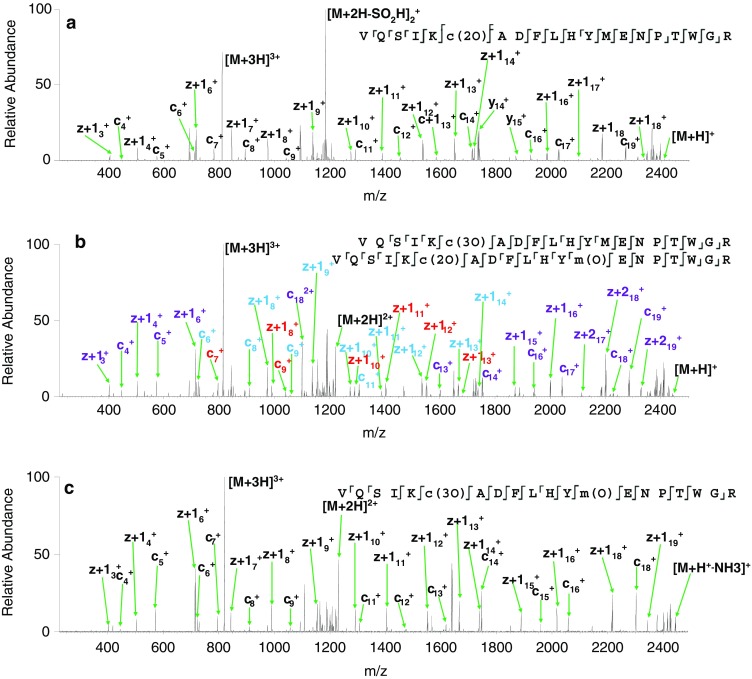
(**a**) ETD MS/MS spectrum of 2+ ions of [VQSIKCADFLHYMENPTWGR +2O]; (**b**) ETD MS/MS fragmentation of 3+ ions of [VQSIKCADFLHYMENPTWGR +3O]. Fragments shown in purple can belong to either species; fragments shown in red belong to the species with two oxidations on the cysteine and one on the methionine; fragments shown in blue belong to the species with three oxidations on the cysteine; (**c**) CID MS/MS fragmentation of the quadruple oxidation product of VQSIKCADFLHYMENPTWGR. Observed fragments are summarized on the peptide sequences, inset. Lower case denotes modified amino acid residues

Figure 1 shows the mass spectrum obtained following SCW hydrolysis of the peptide at 140 °C for 10 min. The most intense peak was observed at *m*/*z* 809.7109 and corresponds to triply protonated ions of peptide VQSIKCADFLHYMENPTWGR plus two oxygen atoms (*m*/*z*_calc_ 809.7122). (Low abundance doubly protonated ions of this species were also observed at *m*/*z* 1214.0621 (*m*/*z*_calc_ 1214.0646). Figure 2a shows the ETD MS/MS spectrum of the 3+ ions and the c and z fragments are summarized in Supplementary Table 2. Manual analysis of the mass spectrum revealed that both oxidations occur on the cysteine residue (i.e., sulfinic acid is formed). There are a number of peaks that correspond to amino acid side-chain losses. These fragments are commonly observed in electron-mediated dissociation [4]. Of particular note here is the peak corresponding to loss of the sulfinic acid side chain (–SO_2_H_2_) observed at *m*/*z* 1181.0746 (*m*/*z*_calc_ 1181.0756), which confirms the double oxidation on cysteine.

**Figure 3:**
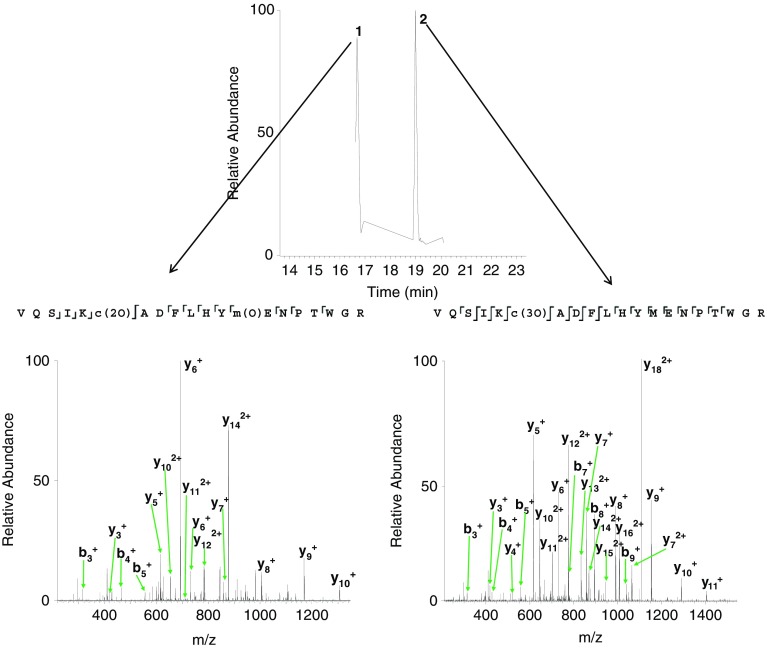
Extracted ion chromatogram (*m*/*z* 815.0426, [VQSIKCADFLHYMENPTWGR +3O]) obtained following LC CID MS/MS and the two corresponding CID MS/MS spectra at retention times 16 min 45 s and 19 min. Observed fragments are summarized on the peptide sequences, inset. Lower case denotes modified amino acid residues

The peak at *m*/*z* 815.0426 (Figure 1) corresponds to triply protonated ions of the peptide plus three oxygen atoms (*m*/*z*_calc_ 815.0438). This peak was isolated and fragmented by use of ETD. Peaks corresponding to fragments from both triply oxidated cysteine (i.e., sulfonic acid) and doubly oxidated cysteine (sulfinic acid) together with methionine oxidation, were observed (Figure 2b), suggesting two species were present. Loss of both the sulfinic acid side chain (*m*/*z* 1189.0718) and (low abundance) sulfonic acid side chains were observed (*m*/*z* 1181.0777) in the +2 charge state (*m*/*z*_calc_ 1189.0731 and 1181.0756). LC CID MS/MS was performed and two species were seen to elute at retention times of ~16 min 45 s and ~19 min (Figure 3). CID MS/MS of the species eluting at RT ~16 min 45 s reveals the addition of two oxygen atoms on the cysteine residue (formation of sulfinic acid) and one oxygen atom to the methionine residue (Supplementary Table 3i). CID MS/MS of the species eluting at RT 19 min shows that all three oxidations occur on the cysteine residue, forming sulfonic acid (Supplementary Table 3ii). The oxidation of methionine is expected as numerous studies have shown methionine to be readily oxidized to methionine sulfoxide [5].

A peak corresponding to 3+ ions of the peptide plus four oxygen atoms was observed at *m*/*z* 820.3743 (*m*/*z*_calc_ 820.3754) (Figure 1). These ions were isolated and fragmented by ETD to reveal a single species comprising three oxidations of the cysteine residue and a single oxidation of the methionine residue (Supplementary Table 4 and Figure 2c).

The only peak corresponding to a SCW cleavage product was observed at *m*/*z* 517.5779 (*m*/*z*_calc_ 517.5785) and corresponds to FLHYMENPTWGR. This assignment was confirmed by CID (data not shown). The peptide product is the result of cleavage C-terminal to the aspartic acid residue in the original peptide, consistent with our previous work, which found aspartic acid to be the most common site of SCW-induced cleavage [2]. Oxidation of this peptide product was also observed at *m*/*z* 522.9094 (*m*/*z*_calc_ 522.9105). CID MS/MS analysis revealed methionine to be the site of oxidation (data not shown).

SCW treatment was also performed at 160 °C, 180 °C, and 200 °C (Table 1 and Supplementary Figures 2–4). In each case, the residency time was 10 min. A greater amount of peptide bond hydrolysis was observed as the temperature increased, as expected from our earlier work [2]. Following treatment at 160 °C, the cleavage product FLHYMENPTWGR represents the base peak in the mass spectrum. At SCW conditions of 180 °C, water loss could also be detected as a modification. In our previous study, we showed that inclusion of water loss as a dynamic modification in the automated protein database search of LC MS/MS data obtained from SCW hydrolysates resulted in a 9% increase in peptide identifications for α-globin, β-globin, BSA, and β-casein at SCW conditions of 160 °C (0 min), 160 °C (20 min), and 207 °C (20 min). In addition Basil et al. showed that in the thermal decomposition of peptides at comparable temperatures to those used here, dehydration products could be detected [6]. We were unable to identify the specific sites of water loss: CID is not a reliable indicator as the CID process itself can result in water loss and ETD did not produce a complete set of fragment ions. Interestingly, Basil et al. further identify C-terminal amidation as a modification through thermal denaturation. We observe a small amount of this modification under the two harshest SCW conditions (180 °C and 200 °C): CID MS/MS was used to confirm that the amidation occurred on the C-terminus (Supplementary Figure 5 and Supplementary Table 5).

**Figure 4:**
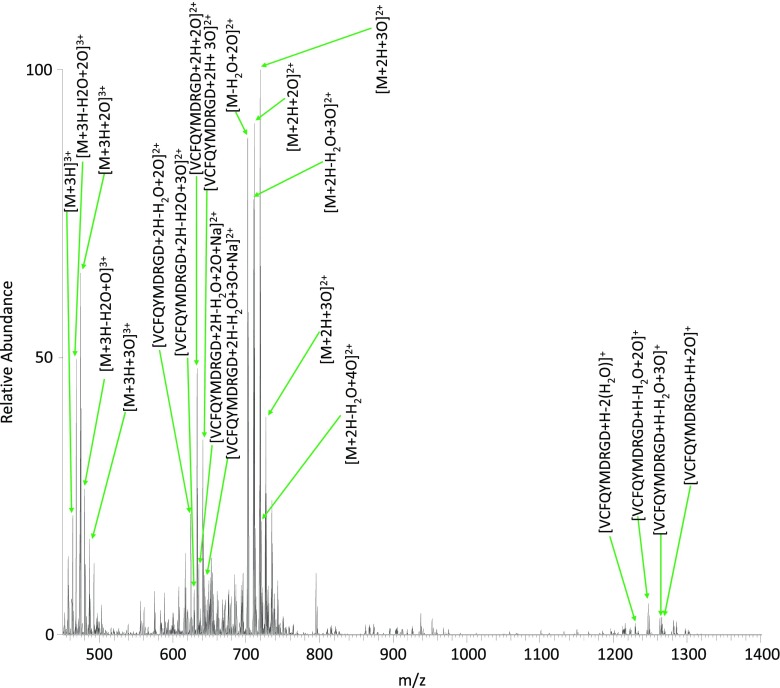
Direct infusion electrospray mass spectrum of peptide VCFQYMDRGDR treated with SCW at 140 °C for 10 min

**Figure 5:**
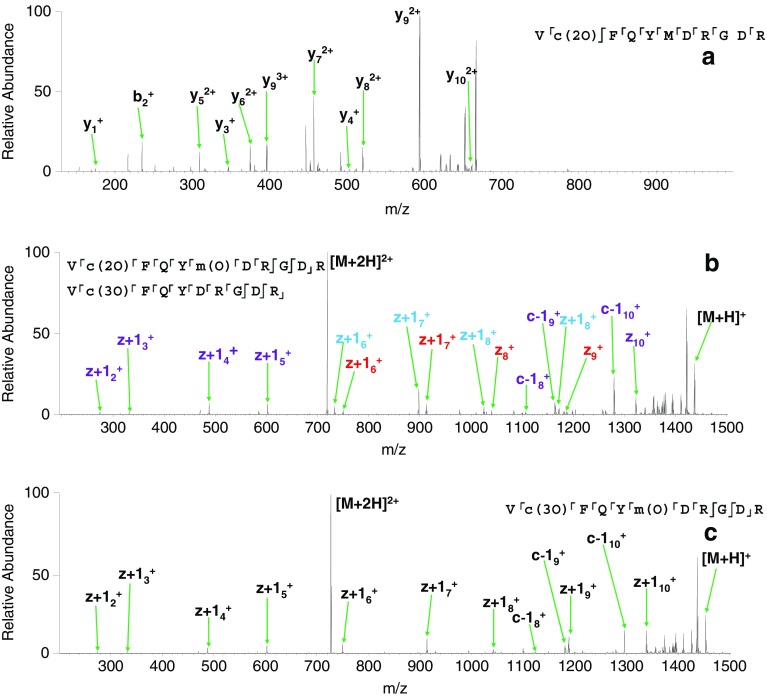
(**a**) CID MS/MS spectrum of 3+ ions of [VCFQYMDRGDR +2O]; (**b**) ETD MS/MS fragmentation of 3+ ions of [VCFQYMDRGDR +3O]. Fragments shown in purple belong to either species; fragments shown in red belong to the species with two oxidations on the cysteine and one on the methionine; fragments shown in blue belong to the species with three oxidations on the cysteine; (**c**) ETD MS/MS spectrum of 2+ ions of [VCFQYMDRGDR +3O]. Observed fragments are summarized on the peptide sequences, inset. Lower case denotes modified amino acid residues

A second peptide that also contained cysteine and methionine, VCFQYMDRGDR, was treated with subcritical water at 140 °C for 10 min. The direct infusion electrospray mass spectrum of the peptide prior to subcritical treatment is shown in Supplementary Figure 6, with peak assignments detailed in Supplementary Table 6. Peaks at *m*/*z* 469.2041 and 703.3028 correspond to singly oxidized species (*m*/*z*_calc_ 469.2044 and 703.3030), which CID MS/MS confirmed as methionine oxidation. Figure 4 shows the direct infusion electrospray mass spectrum of the SCW hydrolysate (see also Table 2). As observed for VQSIKCADFLHYMENPTWGR, the most intense peaks correspond to oxidized forms of the peptide. Peaks observed at *m*/*z* 474.5359 (+3) and *m*/*z* 711.3004 (+2) correspond to the peptide plus the addition of two oxygen atoms (*m*/*z*_calc_ 474.5360 and 711.3000). CID MS/MS analysis of the 3+ ions confirmed that the oxidation occurs solely on the cysteine residue (Figure 5a and Supplementary Table 7).

**Table 2:** Ions Observed Following SCW Hydrolysis of VCFQYMDRGDR at 140 °C for 10 min

*m*/*z*	z	Calculated mass (Da)	Measured mass (Da)	Peptide	ΔPPM
463.8730	3	1388.5965	1388.5972	VCFQYMDRGDR	0.5
468.5323	3	1402.5758	1402.5751	VCFQYMDRGDR (–H_2_O+2O)	-0.5
473.8640	3	1418.5707	1418.5702	VCFQYMDRGDR (–H_2_O+3O)	–0.4
474.5359	3	1420.5863	1420.5859	VCFQYMDRGDR (+2O)	–0.3
479.8675	3	1436.5812	1436.5807	VCFQYMDRGDR (+3O)	–0.4
624.2444	2	1246.4747	1246.4742	VCFQYMDRGD (–H_2_O+2O)	–0.3
632.2418	2	1262.4696	1262.4690	VCFQYMDRGD (–H_2_O+3O)	–0.4
633.2498	2	1264.4852	1264.4850	VCFQYMDRGD (+2O)	–0.1
635.2353	2	1268.4561	1268.4560	VCFQYMDRGD (–H_2_O+2O+Na)	0.0
641.2472	2	1280.4811	1280.4798	VCFQYMDRGD (+3O)	–1.0
643.2327	2	1284.4510	1284.4508	VCFQYMDRGD (–H2O+3O+Na)	–0.1
652.2381	2	1302.4625	1302.4616	VCFQYMDRGD (+3O+Na)	–0.7
702.2950	2	1402.5758	1402.5754	VCFQYMDRGDR (–H_2_O+2O)	–0.2
710.2924	2	1418.5707	1418.5702	VCFQYMDRGDR (–H_2_O+3O)	–0.3
711.3004	2	1420.5863	1420.5862	VCFQYMDRGDR (+2O)	–0.1
718.2898	2	1434.5656	1434.5650	VCFQYMDRGDR (–H_2_O+4O)	–0.4
719.2999	2	1436.5812	1436.5852	VCFQYMDRGDR (+3O)	2.8
727.2950	2	1452.5761	1452.5754	VCFQYMDRGDR (+4O)	–0.5
1229.4707	1	1228.4641	1228.4634	VCFQYMDRGD (–2H_2_O+2O)	–0.6
1247.4811	1	1246.4747	1246.4738	VCFQYMDRGD (–H_2_O+2O)	–0.7
1263.4760	1	1262.4696	1262.4687	VCFQYMDRGD (–H_2_O+3O)	–0.7
1265.4926	1	1264.4852	1264.4853	VCFQYMDRGD (+2O)	0.1
1281.4868	1	1280.4811	1280.4795	VCFQYMDRGD (+3O)	–1.2
1285.4578	1	1284.4510	1284.4505	VCFQYMDRGD (–H_2_O+3O+Na)	–0.3

**Figure 6:**
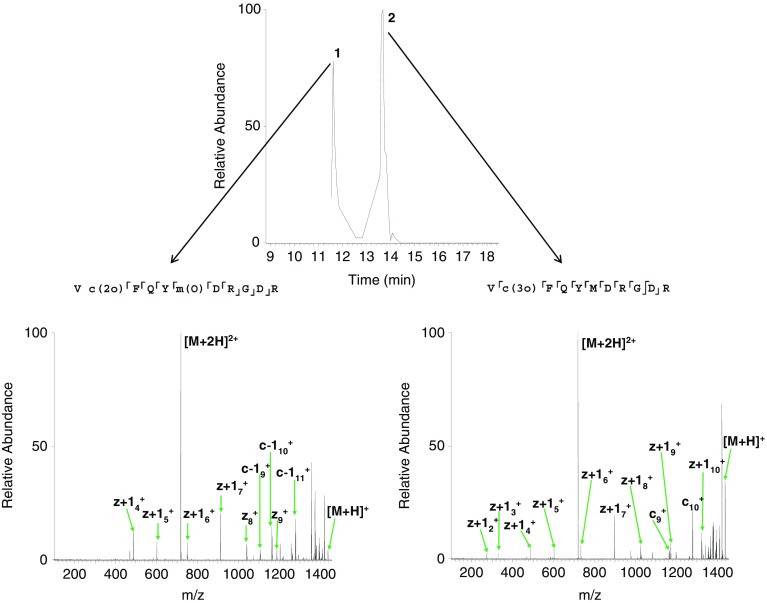
Extracted ion chromatogram (*m*/*z* 719.2973, [VCFQYMDRGDR +3O]) obtained following LC ETD MS/MS and the two corresponding ETD MS/MS spectra at retention times 11 min 30 s and 13 min 30 s. Observed fragments are summarized on the peptide sequences, inset. Lower case denotes modified amino acid residues

Peaks observed at *m*/*z* 479.8675 (+3) and *m*/*z* 719.2999 (+2) (*m*/*z*_calc_ 479.8677 and 719.2979) (Figure 4) correspond to the peptide plus the addition of three oxygen atoms. Subsequent MS/MS analysis proved ambiguous, i.e., fragments from both Vc(3O)FQYMDRGDR and Vc(2O)FQYm(O)DRGDR were identified (Figure 5b). Furthermore, peaks corresponding to the loss of sulfinic acid (*m*/*z* 1371.6102), sulfonic acid (*m*/*z* 1355.6148), and methionine sulfoxide (*m*/*z* 1374.5942) side chains were observed (*m*/*z*_calc_ 1371.6099, 1355.6150, and 1374.5969). LC ETD MS/MS was performed and two species were seen to elute at retention times of ~11.5 min and ~13.5 min. ETD MS/MS of the species eluting at ~11.5 min revealed the presence of Vc(2O)FQYm(O)DRGDR and ETD MS/MS of the species eluting at ~13.5 min revealed the presence of Vc(3O)FQYMDRGDR (Figure 6 and Supplementary Table 8). Furthermore, the loss of the sulfinic acid side chain (*m*/*z*_meas_ 1371.6091, *m*/*z*_calc_ 1371.6099) was only observed in the ETD mass spectrum obtained at RT 11.5 min, and the loss of the sulfonic acid side chain (*m*/*z*_meas_ 1355.6128, *m*/*z*_calc_ 1355.6150) was only observed in the ETD mass spectrum obtained at RT 13.5 min.

The peak at *m*/*z* 727.2950 (Figure 4), corresponding to the addition of four oxygen atoms (*m*/*z*_calc_ 727.2953), was isolated and fragmented by ETD MS/MS to reveal the addition of three oxygen atoms on the cysteine residue and the addition of one oxygen atom on the methionine residue (Figure 5c and Supplementary Table 9).

In addition to oxidation, extensive dehydration was observed for this peptide. We attribute this to the presence of two aspartic acid residues in the peptide sequence. Dehydration of the doubly oxidized species was observed (*m*/*z*_meas_ 468.5323 (+3) and 702.2950 (+2), *m*/*z*_calc_ 468.5322 and 702.2496), as was dehydration of the triply oxidized species [*m*/*z*_meas_ 473.8640 (+3) and 710.2924 (+2), *m*/*z*_calc_ 473.8638 and 710.2921]. ETD MS/MS was performed; however, the site of water loss was ambiguous in all cases.

As with the previous peptide, cleavage at the C-terminal of the aspartic acid was also observed. Peaks were observed corresponding to the addition of two oxygen atoms with (*m*/*z* 624.2444) and without water loss (*m*/*z* 633.2498) (*m*/*z*_calc_ 624.2446 and 633.2499), and addition of three oxygen atoms with (*m*/*z* 632.2418) and without water loss (*m*/*z* 641.2472) (*m*/*z*_calc_ 632.2421 and 641.2471).

The results above show that the most commonly occurring amino acid side-chain modifications following treatment with SCW are oxidation of cysteine and methionine residues. In order to determine whether other modifications might occur in the absence of those residues, SCW treatment was performed on (1) the peptide VQSIKCADFLHYMENPTWGR following capping of the cysteine residue, and (2) a model peptide VQSIKADFLHYENPTWGR that did not contain either cysteine or methionine.

Supplementary Figure 7 shows the direct infusion electrospray mass spectrum of the iodoactamide-treated peptide VQSIKCADFLHYMENPTWGR prior to SCW treatment (see also Supplementary Table 10). CID MS/MS confirms carbamidomethylation of the cysteine residue (Supplementary Figure 8 and Supplementary Table 11).

**Figure 7:**
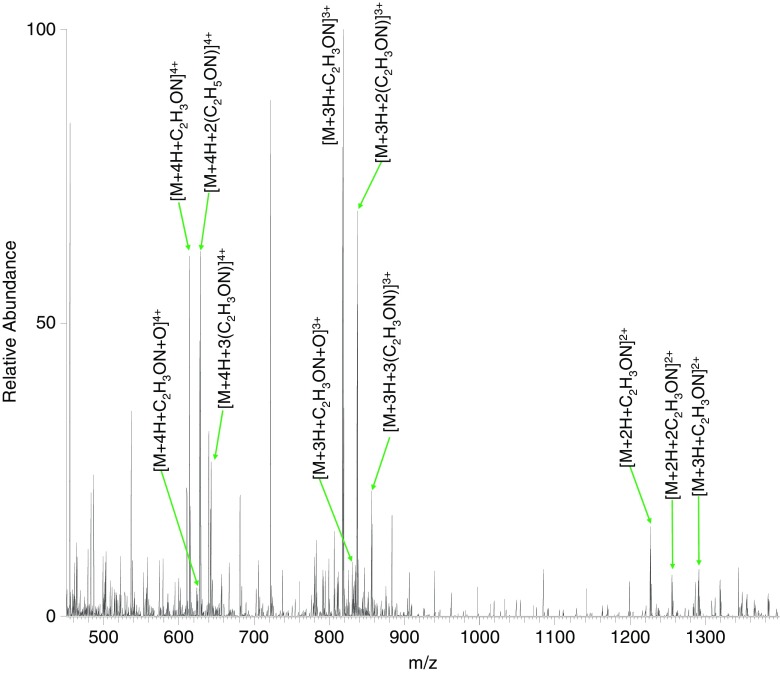
Direct infusion electrospray mass spectrum of peptide VQSIKCADFLHYMENPTWGR following iodoacetamide treatment treated with SCW at 140 °C for 10 min

Figure 7 shows the direct infusion electrospray mass spectrum of the resulting mixture when the peptide VQSIKCADFLHYMENPTWGR pretreated with iodoacetamide was subjected to SCW treatment at 140 °C for 10 min (see also Table 3). The most abundant multiply charged ions are the carbamidomethylated peptide ions in the 3+ charge state, observed at *m*/*z* 818.0517 (*m*/*z*_calc_ 818.0561). These species were also observed in the 4+ charge state (*m*/*z*_meas_ 613.7906; m/z_calc_ 613.7939). In addition, a single oxidation was seen to occur at *m*/*z* 617.7893 (+4) and 823.3833 (+3) (*m*/*z*_calc_ 617.7926 and 823.3877). The triply protonated species was fragmented by ETD, revealing that oxidation occurred on the methionine residue (Supplementary Figure 9 and Supplementary Table 12). The peak at *m*/*z* 1190.0732 (+2) corresponds to loss of the carbamidomethylated cysteine side chain (*m*/*z*_calc_ 1190.0737).

**Table 3:** Ions Observed Following SCW Hydrolysis of Iodoacetamice Pretreated VQSIKCADFLHYMENPTWGR at 140 °C for 10 min

*m*/*z*	z	Calculated mass (Da)	Measured mass (Da)	Peptide	ΔPPM
454.8248	1			Singly charged (unassigned)	
613.7906	4	2451.1464	2451.1333	VQSIKCADFLHYMENPTWGR (+ C_2_H_3_ON)	–5.3
617.7893	4	2467.1413	2467.1281	VQSIKCADFLHYMENPTWGR (+ C_2_H_3_ON + O)	–5.3
628.0459	4	2508.1678	2508.1545	VQSIKCADFLHYMENPTWGR (+2(C_2_H_3_ON))	–5.3
642.3011	4	2565.1893	2565.1753	VQSIKCADFLHYMENPTWGR (+3(C_2_H_3_ON))	–5.5
721.664	1			Singly charged (unassigned)	
818.0517	3	2451.1464	2451.1333	VQSIKCADFLHYMENPTWGR (+ C_2_H_3_ON)	–5.3
823.3833	3	2467.1413	2467.1281	VQSIKCADFLHYMENPTWGR (+ C_2_H_3_ON + O)	–5.4
837.0587	3	2508.1678	2508.1543	VQSIKCADFLHYMENPTWGR (+2(C_2_H_3_ON))	–5.4
856.0685	3	2565.1893	2565.1837	VQSIKCADFLHYMENPTWGR (+3(C_2_H_3_ON))	–2.2
1226.5742	2	2451.1464	2451.1338	VQSIKCADFLHYMENPTWGR (+ C_2_H_3_ON)	–5.1
1255.0846	2	2508.1678	2508.1546	VQSIKCADFLHYMENPTWGR (+2(C_2_H_3_ON))	–5.3
1283.5925	2	2565.1893	2565.1704	VQSIKCADFLHYMENPTWGR (+3(C_2_H_3_ON))	–7.4

Interestingly, we observe the addition of a further carbamidomethyl groups to the peptide at *m*/*z* 628.0459 (+4), 837.0587 (+3), 1255.0846 (+2) (*m*/*z*_calc_ 628.0492, 837.0632, and 1255.0912), as well as species with three carbamidomethyl groups at *m*/*z* 642.3011 (+4), 837.0587 (+3), and 1283.5925 (+2) (*m*/*z*_calc_ 642.3046, 837.0704, and 1283.6019). That is, the excess iodoacetamide present in the sample further alkylates the peptide under SCW conditions. Alkylation of cysteine by iodoacetamide occurs via nucleophilic substitution (SN_2_) at basic pH. It is also known that under certain conditions (pH, concentration, length of incubation), alkylation of other amino acid residues (methionine, histidine, lysine, tyrosine, glutamic acid, and aspartic acid) and the N-terminus or C-terminus by iodoacetamide can occur [7–11]. Our data suggest that SCW promotes substitution by other nucleophiles. It was not possible to determine the sites of modification due to the composite MS/MS spectra obtained, even when coupled with liquid chromatography (data not shown).

The presence of nonspecific carbamidomethylation could be detrimental for proteomics analysis and therefore we investigated the effect of quenching excess iodoacetamide with dithiothreitol [12] prior to SCW treatment. Supplementary Figure 10 shows the direct infusion electrospray mass spectrum of the iodoacetamide and DTT-treated peptide VQSIKCADFLHYMENPTWGR before and after SCW treatment at 140 °C for 10 min. Both spectra are dominated by singly charged ions, likely due to the excess of both iodoacetamide and dithiothreitol. The singly carbamidomethylated peptide is observed at *m*/*z* 818.0562 (+3) and 1226.5809 (+2), and there is no evidence for multiple carbamidomethylation. Peaks corresponding to addition of hydrogen and iodine to carbamidomethylated peptides are observed in both the non-SCW treated sample at *m*/*z* 1290.5372 and *m*/*z* 1354.4932, and the SCW treated sample at *m*/*z* 1290.5365 and *m*/*z* 1354.4926 (*m*/*z*_calc_ 1290.5366 and 1354.4928). These modifications were not observed in the non-DTT treated sample. Furthermore, methionine oxidation was not observed under these conditions.

**Figure 8:**
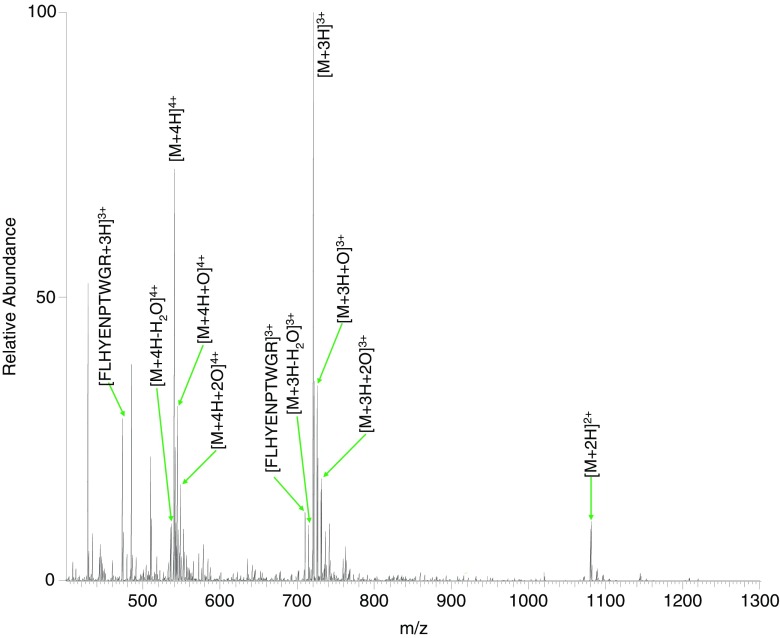
Direct infusion electrospray mass spectrum of peptide VQSIKADFLHYENPTWGR treated with SCW at 140 °C for 10 min

To further probe the effects of SCW hydrolysis on residues that were not cysteine or methionine, a synthetic peptide was designed, VQSIKADFLHYENPTWGR (Supplementary Figure 11 and Supplementary Table 13). The peptide VQSIKADFLHYENPTWGR was treated with SCW at 140 °C for 10 min. Direct infusion MS revealed that the most abundant species was the unmodified peptide in the +3 charge state (Figure 8 and Table 4), in contrast to SCW-treated VQSIKCADFLHYMENPTWGR, in which the major product was an oxidized form of the peptide.

**Table 4:** Ions Observed Following SCW Hydrolysis of VQSIKADFLHYENPTWGR at 140 °C for 10 min

*m*/*z*	z	Calculated mass (Da)	Measured mass (Da)	Peptide	ΔPPM
473.9003	3	1418.6731	1418.6791	FLHYENPTWGR	4.2
536.5265	4	2142.0647	2142.0769	VQSIKADFLHYENPTWGR (–H_2_O)	5.7
541.0280	4	2160.0752	2160.0829	VQSIKADFLHYENPTWGR	3.6
545.0272	4	2176.0701	2176.0797	VQSIKADFLHYENPTWGR (+O)	4.4
549.0260	4	2192.0650	2192.0749	VQSIKADFLHYENPTWGR (+2O)	4.5
710.3466	2	1418.6731	1418.6786	FLHYENPTWGR	3.9
715.0316	3	2142.0647	2142.0730	VQSIKADFLHYENPTWGR (–H_2_O)	3.9
721.0348	3	2160.0752	2160.0826	VQSIKADFLHYENPTWGR	3.4
726.3670	3	2176.0701	2176.0792	VQSIKADFLHYENPTWGR (+O)	4.2
731.6989	3	2192.0650	2192.0749	VQSIKADFLHYENPTWGR (+2O)	4.5
1081.0483	2	2160.0752	2160.0820	VQSIKADFLHYENPTWGR	3.2

Peaks observed at *m*/*z* 545.0272 (+4) and *m*/*z* 726.3670 (+3) corresponds to the peptide plus addition of a single oxygen atom (*m*/*z*_calc_ 545.0248 and 726.3640). ETD MS/MS of the ions with *m*/*z* 545.0272 confirmed that oxidation of the tryptophan residue had occurred (Supplementary Figure 12 and Supplementary Table 14). The oxidation of tryptophan has previously been reported in proteomic studies [13, 14]. Peaks corresponding to the addition of two oxygen atoms to the peptide were also observed in the +4 and +3 charge states at *m*/*z* 549.0260 and *m*/*z* 731.6989 (*m*/*z*_calc_ 549.0235 and 731.6956). Analysis of the double oxidation product using CID MS/MS revealed that both oxidations occur on the tryptophan (Supplementary Figure 13 and Supplementary Table 15). This is consistent with work by Taylor et al., which shows tryptophan is able to adopt a second oxidation state in mitochondrial proteins [15]. Dehydration was also observed under these conditions at *m*/*z* 715.0316 (*m*/*z*_calc_ 715.0288). Isolation of the peak and ETD MS/MS showed water loss to occur at the aspartic acid residue (Supplementary Figure 14 and Supplementary Table 16). The loss of water from amino acid residues has previously been investigated by Sun et al., who demonstrated that aspartic acid is a likely candidate [16]. Finally, for this peptide, an SCW hydrolysis cleavage product was observed at *m*/*z* 710.3466, corresponding to the peptide, FLHYENPTWGR (*m*/*z*_calc_ 710.3438).

In light of the above results, the data obtained from the SCW treatment of proteins in our original publication [2] were re-analyzed. The data were searched against the relevant protein sequence as obtained from UniProt using the SEQUEST algorithm in Proteome Discoverer ver. 1.4.1.14 (Thermo Fisher Scientific). Data were searched using “nonspecific enzyme.” Precursor tolerance was 10 ppm and MS/MS tolerance was 0.5 Da. The following were allowed as dynamic modifications: addition of two and three oxygen atoms on cysteine, addition of one and two oxygen atoms on tryptophan, water loss from aspartic acid residues, C-terminal amidation and methionine oxidation. (Note that methionine oxidation was also allowed as dynamic modification in the previous analysis). The database search resulted in an increase in the number of identified peptides. An additional 108 (that is, an increase of 63.1%), 121 (38.7%), and 64 (34.2%) peptides were identified for α-globin hydrolyzed at 160 °C for 0 min, 160 °C for 20 min, and 207 °C for 20 min. A further 69 (increase of 24.3%), 96 (31.1%), and 74 (48.7%) peptides were identified in β-globin hydrolyzed at 160 °C for 0 min, 160 °C for 20 min, and 207 °C for 20 min. Twenty-eight (28.3%), 91 (37.0%), and 58 (25.3%) further peptides were identified for BSA hydrolyzed at 160 °C for 0 min, 160 °C for 20 min, and 207 °C for 20 min. Thirteen (9%), 163 (25.2%), and 168 (23.5%) additional peptides were identified for β-casein hydrolyzed at 160 °C for 0 min, 160 °C for 20 min, and 207 °C for 20 min.

## Conclusion

The results show that SCW hydrolysis of peptides results in efficient oxidation of the hydrolysates. SCW treatment under mild conditions (140 °C for 10 min) resulted in oxidation of cysteine and methionine residues. Oxidation of cysteine to sulfinic and sulfonic acid was observed. SCW treatment of a peptide that did not contain cysteine or methionine resulted in oxidation of tryptophan. Under harsher SCW conditions (160 °C–180 °C), dehydration and amidation of the peptides were detected. Water loss occurs at aspartic acid. In addition, the C-terminal of aspartic acid is consistently shown to be a site of preferential cleavage for SCW.

In our previous work, we suggested that SCW hydrolysis has the potential to be an alternative to enzymatic digestion in proteomics experiments. We do not envisage the side-chain modifications identified here that are induced by SCW hydrolysis to be detrimental in the identification of proteins. Existing bottom-up proteomics strategies involve digestion of proteins either in solution or following one- or two-dimensional gel electrophoresis [17]. Oxidation is commonly encountered in cysteine, methionine, and tryptophan residues during gel electrophoresis and associated sample handling [18–20]. Whilst oxidation may result in reduced signal-to-noise due to the spread of signal over a number of mass channels, it does not prevent protein identification.

## Electronic supplementary material

Below is the link to the electronic supplementary material.

ESM 1 (DOCX 21 kb)

ESM 2 (DOCX 78 kb)

ESM 3 (PPTX 372 kb)
