# Supplementary material for: Subcritical Water Hydrolysis of Peptides: Amino Acid Side-Chain Modifications
Source: J Am Soc Mass Spectrom. 2017 May 17;28(9):1775–86. doi: 10.1007/s13361-017-1676-1 (PMC5556142; doi:10.1007/s13361-017-1676-1)
Supplement: Supplementary file 1 — (DOCX 21 kb) [file 13361_2017_1676_MOESM1_ESM.docx]

**Sub-critical water hydrolysis of peptides: Amino acid side-chain modifications**

Thomas Powell^1^, Steve Bowra^2^ and Helen J. Cooper^1^*

^1^School of Biosciences, University of Birmingham, Edgbaston, Birmingham B15 2TT, UK.

^2^ Phytatec (UK) Ltd., Plas Gogerddan, Aberystwyth SY23 3EB, UK

**Supplementary Information**

**Supplementary Figure 1** - Direct infusion electrospray MS of peptide VQSIKCADFLHYMENPTWGR.

**Supplementary Figure 2** - Direct infusion electrospray MS of peptide VQSIKCADFLHYMENPTWGR treated with SCW at 160^o^C for 10 minutes.

**Supplementary Figure 3** - Direct infusion electrospray MS of peptide VQSIKCADFLHYMENPTWGR treated with SCW at 180^o^C for 10 minutes.

**Supplementary Figure 4** - Direct infusion electrospray MS of peptide VQSIKCADFLHYMENPTWGR treated with SCW at 200^o^C for 10 minutes.

**Supplementary Figure 5** - Direct infusion electrospray MS of 3+ ions of CID MS/MS spectrum of 2+ ions of [FLHYMENPT + O + C-term amidation]

**Supplementary Figure 6** - Direct infusion electrospray MS of peptide VCFQYMDRGDR.

**Supplementary Figure 7** - Direct infusion electrospray MS of peptide VQSIKCADFLHYMENPTWGR treated with iodoacetamide.

**Supplementary Figure 8** - CID MS/MS spectrum of 3+ ions of [VQSIKADFLHYENPTWGR+C_2_H_5_ON]

**Supplementary Figure 9** - ETD MS/MS spectrum of 3+ ions of [VQSIKADFLHYENPTWGR+C_2_H_5_ON+O]

**Supplementary Figure 10** - Direct infusion electrospray MS of peptide VQSIKADFLHYENPTWGR treated with iodoacetamide and DTT i) prior to SCW hydrolysis and ii) hydrolysed at 140^o^C for 10 minutes.

**Supplementary Figure 11** - Direct infusion electrospray MS of peptide VQSIKADFLHYENPTWGR.

**Supplementary Figure 12** - ETD MS/MS spectrum of 4+ ions of [VQSIKADFLHYENPTWGR+O]

**Supplementary Figure 13** - CID MS/MS spectrum of 4+ ions of [VQSIKADFLHYENPTWGR+2O]

**Supplementary Figure 14** - ETD MS/MS spectrum of 3+ ions of [VQSIKADFLHYENPTWGR- H_2_O]

**Supplementary Table 1** - Ions identified from the direct infusion electrospray MS of untreated VQSIKCADFHYMENPTWGR.

**Supplementary Table 2** - Peak assignments following ETD MS/MS of *m/z* 809.7109

**Supplementary Table 3** – Peak assignments following LC CID MS/MS of *m/z* 815.0426 at i) RT ~16 min 45 s and ii) ~19 min.

**Supplementary Table 4** - Peak assignments following CID MS/MS of *m/z* 820.3787

**Supplementary Table 5** - Peak assignments following CID MS/MS of *m/z* 583.7703

**Supplementary Table 6** - Ions identified from the direct infusion electrospray MS of untreated VCFQYMDRGDR.

**Supplementary Table 7** - Peak assignments following CID MS/MS of *m/z* 474.5359

**Supplementary Table 8** – Peak assignments following LC ETD MS/MS of *m/z* 719.2973 at i) RT ~11min 30s and ii) ~13 min 30s.

**Supplementary Table 9** – Peak assignments following ETD MS/MS of *m/z* 727.2950

**Supplementary Table 10** - Ions identified from the direct infusion electrospray MS of iodoacetamide treated VQSIKCADFHYMENPTWGR.

**Supplementary Table 11** - Peak assignments following CID MS/MS of *m/z* 818.0553

**Supplementary Table 12** - Peak assignments following ETD MS/MS of *m/z* 823.3833

**Supplementary Table 13** - Ions identified from the direct infusion electrospray MS of untreated VQSIKADFHYENPTWGR.

**Supplementary Table 14** - Peak assignments following ETD MS/MS of *m/z* 545.0272

**Supplementary Table 15** - Peak assignments following CID MS/MS of *m/z* 549.0260

**Supplementary Table 16** - Peak assignments following CID MS/MS of *m/z* 715.0316
