# Supplementary material for: Subcritical Water Hydrolysis of Peptides: Amino Acid Side-Chain Modifications
Source: J Am Soc Mass Spectrom. 2017 May 17;28(9):1775–86. doi: 10.1007/s13361-017-1676-1 (PMC5556142; doi:10.1007/s13361-017-1676-1)
Supplement: Supplementary file 2 — (DOCX 78 kb) [file 13361_2017_1676_MOESM2_ESM.docx]

***m/z* z Calculated mass (Da) Measured mass (Da) Peptide ΔPPM**

599.5376 4 2394.1249 2394.1213 VQSIKCADFLHYMENPTWGR -1.5

609.0241 4 2432.0803 2432.0673 VQSIKCADFLHYMENPTWGR (+K) -5.3

799.0474 3 2394.1249 2394.1204 VQSIKCADFLHYMENPTWGR -1.9

806.3746 3 2416.1063 2416.1020 VQSIKCADFLHYMENPTWGR (+Na) -1.8

811.6962 3 2432.0803 2432.0668 VQSIKCADFLHYMENPTWGR (+K) -5.5

1198.0666 2 2394.1249 2394.1186 VQSIKCADFLHYMENPTWGR -2.6

1209.0577 2 2416.1063 2416.1008 VQSIKCADFLHYMENPTWGR (+Na) -2.3

**Supplementary Table 1** - Ions identified from the direct infusion electrospray MS of untreated VQSIKCADFHYMENPTWGR.

**c**   **c- 1**  **y**  **z+1**

**Calculated Measured ΔPPM Calculated Measured ΔPPM**  **Calculated Measured ΔPPM Calculated Measured ΔPPM**

***m/z* *m/z***  ***m/z* *m/z***  ***m/z* *m/z***  ***m/z* *m/z***

117.1022 116.0944 1 V 20 ---

245.1608 244.1530 2 Q 19 2328.0487 2313.0378 2313.0305 -3.1

332.1928 331.1850 3 S 18 2199.9901 2184.9792 2184.9718 -3.4

445.2769 445.2767 -0.5 444.2691 4 I 17 2112.9581 2097.9472 2097.9435 -1.7

573.3719 573.3716 -0.5 572.3640 5 K 16 1999.8740 1984.8631 1984.8591 -2.0

708.3660 708.3708 6.9 707.3581 6 c (2O) 15 1871.7790 1871.7778 -0.6 1856.7681

779.4031 779.4075 5.7 778.3952 7 A 14 1736.7850 1736.7795 -3.2 1721.7741 1721.7668 -4.2

894.4350 894.4342 -4.8 893.4222 8 D 13 1665.7478 1650.7369 1650.7325 -2.7

1041.4984 1041.5027 4.2 1040.4906 9 F 12 1550.7209 1535.7100 1535.7070 -2.0

1154.5825 1153.5747 10 L 11 1403.6525 1388.6416 1388.6401 -1.1

1291.6414 1291.6453 3.1 1290.6336 11 H 10 1290.5684 1275.5575 1275.5565 -0.8

1454.7047 1454.7082 2.4 1453.6969 12 Y 9 1153.5095 1138.4986 1138.4974 -1.1

1585.7452 1584.7374 1584.7379 0.3 13 M 8 990.4462 975.4353 975.4345 -0.8

1714.7878 1714.7876 -0.1 1713.7800 14 E 7 859.4057 844.3948 844.3942 -0.7

--- 15 N 6 730.3631 715.3522 715.3521 -0.1

1925.8835 1925.8799 -1.8 1924.8757 16 P 5 616.3202 --- --- ---

2026.9312 2026.9268 -2.1 2025.9234 17 T 4 519.2674 504.2565 504.2562 -0.6

2213.0105 2212.0027 18 W 3 418.2197 403.2088 403.2086 -0.5

2270.0320 2270.0223 -4.3 2269.0241 19 G 2 232.1404 217.1295

--- 20 R 1 175.1190 160.1081

**Supplementary Table 2** - Peak assignments following ETD MS/MS of *m/z* 809.7109

**b**  **y**  **y^+2^**

**Calculated Measured ΔPPM**  **Calculated Measured ΔPPM Calculated Measured ΔPPM**

***m/z* *m/z***  ***m/z* *m/z***  ***m/z* *m/z***

--- 1 V 20  --- ---

228.1343 2 Q 19 2344.0515 1172.5297

315.1663 315.1671 2.5 3 S 18 2215.9929 1108.5004

428.2504 428.2509 1.2 4 I 17 2128.9609 1064.9844

556.3453 556.3457 0.7 5 K 16 2015.8768 1008.4423

691.3443 691.3447 2.3 6 c (2O) 15 1887.7818 944.3948

762.3814 7 A 14 1752.7809 876.8944 876.8939 -0.5

877.4084 8 D 13 1681.7437 841.3758

1024.4768 9 F 12 1566.7168 783.8623 783.8618 -0.7

1137.5608 10 L 11 1419.6484 710.3281 710.3271 -1.4

1274.6198 11 H 10 1306.5643 1306.5649 0.5 653.7861 653.7853 -1.2

1437.6831 12 Y 9 1169.5054 1169.5048 -0.5 585.2566

1568.7236 13 m(O) 8 1006.4421 1006.4405 -1.6 503.7250

1697.7662 14 E 7 859.4057 859.4059 0.2 430.2065

1811.8091 15 N 6 730.3631 730.3633 0.3 365.6852

1908.8619 16 P 5 616.3202 616.3208 1.0 308.6637

2009.9095 17 T 4 519.2674 260.1373

2195.9888 18 W 3 418.2197 418.2205 2.0 209.6135

2253.0103 19 G 2 232.1404 116.5738

--- 20 R 1 175.1190 88.0631

**Supplementary Table 3i** – Peak assignments following LC CID MS/MS of *m/z* 815.0426 at RT ~16 min 45 s.

**b**  **y**  **y^+2^**

**Calculated Measured ΔPPM**  **Calculated Measured ΔPPM Calculated Measured ΔPPM**

***m/z* *m/z***  ***m/z* *m/z***  ***m/z* *m/z***

--- 1 V 20  --- ---

228.1343 2 Q 19 2344.0515 1172.5297

315.1663 315.1671 2.5 3 S 18 2215.9929 1108.5004 1108.4980 -2.2

428.2504 428.2513 2.1 4 I 17 2128.9609 1064.9844 1064.9830 -1.7

556.3453 556.3456 0.5 5 K 16 2015.8768 1008.4423 1008.4410 -1.8

707.3422 707.3392 -4.2 6 c (3O) 15 1887.7818 944.3948 944.3935 -1.4

778.3793 778.3765 -3.6 7 A 14 1736.7860 868.8970 868.8963 -0.7

893.4063 893.4036 -3.0 8 D 13 1665.7488 833.3784 833.3782 -0.2

1040.4747 1040.4715 -3.1 9 F 12 1550.7219 775.8649 775.8642 -0.8

1153.5587 10 L 11 1403.6535 1403.6520 -0.1 702.3307 702.3304 -0.4

1290.6177 11 H 10 1290.5694 1290.5660 -2.2 645.7887 645.7882 -0.7

1453.6810 12 Y 9 1153.5105 1153.5080 -1.2 577.2592

1584.7215 13 M 8 990.4472 990.4465 -0.3 495.7276

1713.7641 14 E 7 859.4057 859.4061 0.5 422.2091

1827.8070 15 N 6 730.3631 730.3635 0.5 365.6852

1924.8598 16 P 5 616.3202 616.3204 0.3 308.6637

2025.9074 17 T 4 519.2674 519.2682 1.5 260.1373

2211.9867 18 W 3 418.2197 418.2202 1.2 209.6135

2269.0082 19 G 2 232.1404 116.5738

--- 20 R 1 175.1190 88.0631

**Supplementary Table 3ii** – Peak assignments following LC CID MS/MS of *m/z* 815.0426 at RT ~19 min.

**c**  **z+1**

**Calculated Measured ΔPPM**  **Calculated Measured ΔPPM**

***m/z* *m/z***  ***m/z* *m/z***

117.1022 1 V 20 ---

245.1608 2 Q 19 2345.0316 2345.0199 -5.0

332.1928 3 S 18 2216.9730 2216.9590 -6.3

445.2769 445.2758 -2.5 4 I 17 2129.9410

573.3719 573.3761 7.3 5 K 16 2016.8569 2016.8495 -3.7

724.3658 724.3642 -2.2 6 c (3O) 15 1888.7619 1888.7554 -3.4

795.4029 795.4012 -2.1 7 A 14 1737.7690 1737.7621 -4.0

910.4298 910.4278 -2.2 8 D 13 1666.7318 1666.7242 -4.6

1057.4982 1057.4960 -2.1 9 F 12 1551.7049 1551.6987 -4.0

1170.5823 10 L 11 1404.6365 1404.6420 3.9

1307.6412 1307.6377 -2.7 11 H 10 1291.5524 1291.5487 -2.9

1470.7045 1470.7001 -3.0 12 Y 9 1154.4935 1154.4901 -2.9

1617.7399 1617.7358 -2.5 13 m (O) 8 991.4302 991.4276 -2.6

1746.7825 1746.7744 -4.6 14 E 7 844.3948 844.3929 -2.3

--- 15 N 6 715.3522 715.3509 -1.8

1957.8782 1957.8691 -4.6 16 P 5 ---

2058.9259 2058.9164 -4.6 17 T 4 504.2565 504.2555 -2.0

2245.0052 18 W 3 403.2088 403.2082 -1.5

2302.0267 2302.0086 -7.9 19 G 2 217.1295

--- 20 R 1 160.1081

**Supplementary Table 4** - Peak assignments following ETD MS/MS of *m/z* 820.3743

**b**  **b^+2^**  **y**

**Calculated Measured ΔPPM Calculated Measured ΔPPM**  **Calculated Measured ΔPPM**

***m/z* *m/z***  ***m/z* *m/z***  ***m/z* *m/z***

--- --- 1 F 9 ---

261.1598 --- 2 L 8 1019.4615

398.2187 398.2208 5.3 199.6133 3 H 7 906.3774 906.3836 6.9

561.2820 281.1449 4 Y 6 769.3185 769.3230 5.9

708.3174 708.3216 5.9 354.6626 5 M 5 606.2552

837.3600 837.3647 5.6 419.1839 6 E 4 459.2198

951.4029 951.4080 5.4 476.2054 7 N 3 330.1772 330.1789 5.2

1048.4557 524.7318 524.7348 5.8 8 P 2 216.1343 216.1354 5.2

--- --- 9 t(amidation) 1 119.0815

**Supplementary Table 5** - Peak assignments following CID MS/MS of *m/z* 583.7703

***m/z* z Calculated mass (Da) Measured mass (Da) Peptide ΔPPM**

463.8725 3 1388.5965 1388.5957 VCFQYMDRGDR -0.6

469.2041 3 1404.5914 1404.5905 VCFQYMDRGDR (+O) -0.7

695.3052 2 1388.5965 1388.5958 VCFQYMDRGDR -0.5

703.3028 2 1404.5914 1404.5910 VCFQYMDRGDR (+O) -0.3

706.2962 2 1410.5779 1410.5778 VCFQYMDRGDR (+ Na) 0.0

717.2872 2 1432.5593 1432.5598 VCFQYMDRGDR (+2Na) 0.4

728.2781 2 1454.5407 1454.5416 VCFQYMDRGDR (+3Na) 0.6

**Supplementary Table 6** - Ions identified from the direct infusion electrospray MS of untreated VCFQYMDRGDR.

**b y y^+2^ y^+3^**

**Calculated Measured ΔPPM**  **Calculated Measured ΔPPM Calculated Measured ΔPPM Calculated Measured ΔPPM**

**m/z m/z**  **m/z m/z**  **m/z m/z**  **m/z m/z**

--- **1 V 11** --- --- ---

235.0747 235.0747 0.0 **2 c (2O) 10** 1322.5252 661.7662 661.7665 0.4 441.5133

382.1431 **3 F 9** 1187.5262 594.2667 594.2665 -0.4 396.5136 396.5133 -0.7

510.2017 **4 Q 8** 1040.4578 520.7325 520.7313 -2.4 347.4908

673.2650 **5 Y 7** 912.3992 456.7032 456.7030 -0.5 304.8046

804.3055 **6 M 6** 749.3359 375.1716 375.1713 -0.8 250.4502

919.3324 **7 D 5** 618.2954 309.6513 309.6512 -0.4 206.7700

1075.4335 **8 R 4** 503.2685 503.2682 -0.6 252.1379 168.4277

1132.4550 **9 G 3** 347.1674 347.1671 -0.9 174.0873 ---

1247.4819 **10 D 2** 290.1459 145.5766 ---

--- **11 R 1** 175.1190 175.1189 -0.6 88.0631 ---

**Supplementary Table 7** - Peak assignments following CID MS/MS of *m/z* 474.5359

**c-1 z z+1**

**Calculated Measured ΔPPM Calculated Measured ΔPPM Calculated Measured ΔPPM**

**m/z m/z m/z m/z m/z m/z**

116.0944 1 V 11 --- ---

251.0934 2 c (2O) 10 1322.5014 1323.5092

398.1618 3 F 9 1187.5024 1187.5022 -0.2 1188.5102

526.2204 4 Q 8 1040.4340 1040.4336 -0.4 1041.4418

689.2837 5 Y 7 912.3754 913.3832 913.3820 -1.3

836.3191 6 m(O) 6 749.3121 750.3199 750.3195 -0.5

951.3460 7 D 5 602.2767 603.2845 603.2840 -0.8

1107.4472 1107.4457 -1.4 8 R 4 487.2497 488.2576 488.2571 -1.0

1164.4686 1164.4673 -1.1 9 G 3 331.1486 332.1565

1279.4956 1279.4950 -0.5 10 D 2 274.1272 275.1350

--- 11 R 1 159.1002 160.1081

**Supplementary Table 8i** – Peak assignments following LC ETD MS/MS of *m/z* 719.2973 at RT ~11min 30s

**c c-1 z z+1**

**Calculated Measured ΔPPM Calculated Measured ΔPPM Calculated Measured ΔPPM Calculated Measured ΔPPM**

**m/z m/z m/z m/z m/z m/z m/z m/z**

117.1022 116.0944 1 V 11 --- ---

268.0961 267.0883 2 c (3O) 10 1322.5014 1322.5018 0.3 1323.5092

415.1645 414.1567 3 F 9 1171.5075 1172.5153 1172.5138 -1.3

543.2231 542.2153 4 Q 8 1024.4391 1025.4469 1025.4462 -0.7

706.2864 705.2786 5 Y 7 896.3805 897.3883 897.3876 -0.8

837.3269 836.3191 6 M 6 733.3172 734.3250 734.3245 -0.7

952.3539 951.3460 7 D 5 602.2767 603.2845 603.2841 -0.7

1108.4550 1107.4472 8 R 4 487.2497 488.2576 488.2571 -1.0

1165.4764 1165.4745 -1.6 1164.4686 9 G 3 331.1486 332.1565 332.1561 -1.2

1280.5034 1279.4956 1279.4957 0.1 10 D 2 274.1272 275.1350 275.1349 -0.4

--- --- 11 R 1 159.1002 160.1081

**Supplementary Table 8ii** – Peak assignments following LC ETD MS/MS of *m/z* 719.2973 at RT ~13 min 30s.

**c-1**  **z**  **z+1**

**Calculated Measured ΔPPM**  **Calculated Measured ΔPPM Calculated Measured ΔPPM**

**m/z m/z**  **m/z m/z**  **m/z m/z**

116.0944 1 V 11 --- ---

267.0883 2 c (3O) 10 1338.4963 1338.4951 -0.9 1339.5041

414.1567 3 F 9 1187.5024 1188.5102 1188.5094 -0.7

542.2153 4 Q 8 1040.4340 1041.4418 1041.4414 -0.4

705.2786 5 Y 7 912.3754 913.3832 913.3829 -0.3

852.3140 6 m (O) 6 749.3121 750.3199 750.3195 -0.5

967.3409 7 D 5 602.2767 603.2845 603.2841 -0.7

1123.4421 1123.4415 -0.5 8 R 4 487.2497 488.2576 488.2572 -0.8

1180.4635 1180.4629 -0.5 9 G 3 331.1486 332.1565 332.1562 -0.9

1295.4905 1295.4895 -0.8 10 D 2 274.1272 275.1350 275.1349 -0.4

--- 11 R 1 159.1002 160.1081

**Supplementary Table 9** - Peak assignments following ETD MS/MS of *m/z* 727.2950

***m/z* z Calculated mass (Da) Measured mass (Da) Peptide ΔPPM**

613.7934 4 2451.1464 2451.1445 VQSIKCADFLHYMENPTWGR (+ C_2_H_5_ON) -0.8

619.2887 4 2473.1278 2473.1257 VQSIKCADFLHYMENPTWGR (+ C_2_H_5_ON) + Na -0.8

818.0553 3 2451.1464 2451.1441 VQSIKCADFLHYMENPTWGR (+ C_2_H_5_ON) -0.9

825.3826 3 2473.1278 2473.1260 VQSIKCADFLHYMENPTWGR (+ C_2_H_5_ON) + Na -0.7

1226.579 2 2451.1464 2451.1434 VQSIKCADFLHYMENPTWGR (+ C_2_H_5_ON) -1.2

**Supplementary Table 10** - Ions identified from the direct infusion electrospray MS of iodoacetamide treated VQSIKCADFHYMENPTWGR.

**b**  **y**  **y^+2^**

**Calculated Measured ΔPPM**  **Calculated Measured ΔPPM Calculated Measured ΔPPM**

***m/z* *m/z***  ***m/z* *m/z***  ***m/z* *m/z***

--- 1 V 20 --- ---

228.1343 2 Q 19 2353.0862 1177.0470

315.1663 3 S 18 2225.0276 1113.0177 1113.0187 0.9

428.2504 428.2514 2.3 4 I 17 2137.9956 1069.5017 1069.5031 1.4

556.3453 556.3464 2.0 5 K 16 2024.9115 1012.9597 1012.9607 1.0

716.3541 6 c (+C_2_H_5_ON) 15 1896.8165 1896.8167 0.1 948.9122

787.4140 787.4146 0.7 7 A 14 1736.7850 1736.7866 0.9 868.8964

902.4410 902.4417 0.7 8 D 13 1665.7478 1665.7499 1.3 833.3778

1049.5094 1049.5104 0.9 9 F 12 1550.7209 1550.7223 0.9 775.8644

1162.5934 10 L 11 1403.6525 1403.6550 1.8 702.3302

1299.6524 1299.6535 0.8 11 H 10 1290.5684 1290.5706 1.7 645.7881

1462.7157 1462.7175 1.2 12 Y 9 1153.5095 1153.5113 1.6 577.2587

1593.7562 1593.7575 0.8 13 M 8 990.4462 990.4481 1.9 495.7270

1722.7988 1722.7995 0.4 14 E 7 859.4057 859.4074 2.0 430.2068

1836.8417 1836.8421 0.2 15 N 6 730.3631 730.3645 1.9 365.6855

1933.8945 1933.8955 0.5 16 P 5 616.3202 616.3214 1.9 308.6640

2034.9421 17 T 4 519.2674 519.2686 2.3 260.1376

2221.0214 18 W 3 418.2197 418.2207 2.4 209.6138

2278.0429 19 G 2 232.1404 116.5741

--- 20 R 1 175.1190 88.0634

**Supplementary Table 11** - Peak assignments following CID MS/MS of *m/z* 818.0553

**c**  **z+1**

**Calculated Measured ΔPPM Calculated Measured ΔPPM**

***m/z* *m/z***  ***m/z* *m/z***

117.1022 1 V 20 ---

245.1608 2 Q 19 2354.0693 2354.0515 -7.5

332.1928 3 S 18 2226.0107 2225.9960 -6.6

445.2769 4 I 17 2138.9787 2138.9654 -6.3

573.3719 573.3694 -4.4 5 K 16 2025.8946 2025.8829 -5.8

733.4026 733.3995 -4.2 6 C 15 1897.7996

804.4397 804.4362 -4.3 7 A 14 1737.7690 1737.7594 -5.5

919.4666 919.4627 -4.2 8 D 13 1666.7318 1666.7228 -5.4

1066.5350 1066.5306 -4.1 9 F 12 1551.7049 1551.6972 -5.0

1179.6191 1179.6135 -4.7 10 L 11 1404.6365 1404.6304 -4.3

1316.6780 1316.6725 -4.1 11 H 10 1291.5524 1291.5471 -4.1

1479.7412 1479.7351 -4.2 12 Y 9 1154.4935 1154.4881 -4.7

1626.7767 1626.7691 -4.6 13 m(O) 8 991.4302 991.4259 -4.3

1755.8193 1755.8100 -5.3 14 E 7 844.3948 844.3914 -4.0

--- 15 N 6 715.3522 715.3493 -4.1

1966.9150 1966.9036 -5.8 16 P 5 ---

2067.9627 2067.9499 -6.2 17 T 4 504.2565 504.2543 -4.4

2254.0420 2254.0235 -8.2 18 W 3 403.2088 403.2071 -4.2

2311.0635 2311.0470 -7.1 19 G 2 217.1295

--- 20 R 1 160.1081

**Supplementary Table 12** - Peak assignments following ETD MS/MS of *m/z* 823.3833

***m/z* z Calculated mass (Da) Measured mass (Da) Peptide ΔPPM**

541.0273 4 2160.0752 2160.0801 VQSIKADFLHYENPTWGR 2.3

550.5151 4 2198.0306 2198.0313 VQSIKADFLHYENPTWGR (+K) 0.3

721.0335 3 2160.0752 2160.0787 VQSIKADFLHYENPTWGR 1.6

728.3627 3 2182.0566 2182.0663 VQSIKADFLHYENPTWGR (+Na) 4.4

733.6844 3 2198.0306 2198.0314 VQSIKADFLHYENPTWGR (+K) 0.4

1081.0487 2 2160.0752 2160.0828 VQSIKADFLHYENPTWGR 3.5

**Supplementary Table 13**  - Ions identified from the direct infusion electrospray MS of untreated VQSIKADFHYENPTWGR.

**c**  **z**  **z+1**

**Calculated Measured ΔPPM**  **Calculated Measured ΔPPM Calculated Measured ΔPPM**

***m/z* *m/z***  ***m/z* *m/z***  ***m/z* *m/z***

117.1022 **1 V 18** --- ---

245.1608 245.1598 -4.1 2 Q 17 2061.9903 2062.9981 2062.9825 -7.6

332.1928 332.1914 -4.2 3 S 16 1933.9317 1934.9395 1934.9273 -6.3

445.2769 445.2750 -4.3 4 I 15 1846.8997 1847.9075 1847.8981 -5.1

573.3719 573.3694 -4.4 5 K 14 1733.8156 1734.8234 1734.8144 -5.2

644.4090 644.4062 -4.3 6 A 13 1605.7206 1606.7285 1606.7207 -4.9

759.4359 759.4362 0.4 7 D 12 1534.6835 1535.6914 1535.6844 -4.6

906.5043 906.5003 -4.4 8 F 11 1419.6566 1420.6644 1420.6578 -4.6

1019.5884 1019.5839 -4.4 9 L 10 1272.5882 1273.5960 1273.5907 -4.2

1156.6473 1156.6420 -4.6 10 H 9 1159.5041 1160.5119 1160.5063 -4.8

1319.7106 1319.7063 -3.3 11 Y 8 1022.4452 1022.4407 -4.4 1023.4530

1448.7532 1448.7477 -3.8 12 E 7 859.3819 859.3781 -4.4 860.3897

--- 13 N 6 730.3393 730.3362 -4.2 731.3471

1659.8489 1659.8420 -4.2 14 P 5 --- ---

1760.8966 1760.8907 -3.4 15 T 4 519.2436 519.2412 -4.6 520.2514

1962.9708 1962.9630 -4.0 16 w(O) 3 418.1959 418.1940 -4.5 419.2037

2019.9878 2019.9820 -2.9 17 G 2 232.1166 233.1244

--- 18 R 1 175.0951 176.1030

**Supplementary Table 14** - Peak assignments following ETD MS/MS of *m/z* 545.0272

**b**  **b^+2^**  **b^+3^**  **y**  **y^+2^**  **y^+3^**

**Calculated Measured ΔPPM Calculated Measured ΔPPM Calculated Measured ΔPPM**  **Calculated Measured ΔPPM Calculated Measured ΔPPM Calculated Measured ΔPPM**

***m/z* *m/z***  ***m/z* *m/z***  ***m/z* *m/z***  ***m/z* *m/z***  ***m/z* *m/z***  ***m/z* *m/z***

--- --- --- 1 V 18 --- --- ---

228.1343 228.1333 -4.4 --- --- 2 Q 17 2094.0039 1047.5059 698.6732 698.6695 -5.3

315.1663 315.1659 -1.3 --- --- 3 S 16 1965.9453 983.4766 655.9870 655.9839 -4.7

428.2504 --- --- 4 I 15 1878.9133 939.9606 626.9763

556.3453 278.6766 --- 5 K 14 1765.8292 883.4185 589.2816

627.3824 314.1951 --- 6 A 13 1637.7343 819.3711 546.5833

742.4094 742.4059 -4.7 371.7086 --- 7 D 12 1566.6972 783.8525 522.9043

889.4778 889.4733 -5.1 445.2428 --- 8 F 11 1451.6702 726.3390 726.3356 -4.7 484.562

1002.5619 1002.5576 -4.3 501.7849 --- 9 L 10 1304.6018 652.8048 435.5392

1139.6208 570.3143 570.3113 -5.3 380.5455 10 H 9 1191.5177 596.2628 397.8445

1302.6841 651.846 434.8999 11 Y 8 1054.4588 527.7333 ---

1431.7267 716.3673 716.3679 0.9 477.9141 12 E 7 891.3955 446.2017 ---

1545.7696 773.3887 773.385 -4.8 515.9284 13 N 6 762.3529 762.3492 -4.9 381.6804 ---

1642.8224 821.9151 548.2794 14 P 5 648.3100 648.3071 -4.5 324.6589 ---

1743.8701 872.439 581.9619 581.9588 -5.4 15 T 4 551.2572 276.1325 ---

1961.9392 981.4735 654.6516 16 W 3 450.2095 450.2075 -4.4 225.6087 ---

2018.9606 1009.9842 673.6588 17 G 2 232.1404 232.1394 -4.3 116.5741 ---

--- --- --- 18 R 1 175.1190 88.0634 ---

**Supplementary Table 15** - Peak assignments following CID MS/MS of *m/z* 549.0260

**c**  **z+1**

**Calculated Measured ΔPPM**  **Calculated Measured ΔPPM**

***m/z* *m/z***  ***m/z* *m/z***

117.1022 1 V 18 ---

245.1608 2 Q 17 2028.9927 2028.9880 -2.3

332.1928 3 S 16 1900.9341 1900.9305 -1.9

445.2769 445.2779 2.3 4 I 15 1813.9021 1813.8998 -1.2

573.3719 573.3730 1.9 5 K 14 1700.8180 1700.8164 -0.9

644.4090 644.4102 1.9 6 A 13 1572.7231 1572.7200 -1.9

741.4254 741.4268 2.0 7 d (-H_2_O) 12 1501.6860 1501.6835 -1.6

888.4938 888.4952 1.6 8 F 11 1386.6590

1001.5779 1001.5794 1.5 9 L 10 1257.6011 1257.6013 0.2

1138.6368 1138.6382 1.3 10 H 9 1144.5170 1144.5128 -3.7

1301.7001 1301.7009 0.7 11 Y 8 1007.4581 1007.4598 1.7

1430.7427 1430.7432 0.4 12 E 7 844.3948 844.3971 2.7

--- 13 N 6 715.3522

1641.8384 1641.8367 -1.0 14 P 5 ---

1742.8861 1742.8847 -0.8 15 T 4 504.2565 504.2575 2.0

1928.9654 1928.9595 -3.0 16 W 3 403.2088 403.2097 2.2

1985.9869 1985.9817 -2.6 17 G 2 217.1295 217.1303 3.7

--- 18 R 1 160.1081

**Supplementary Table 16** - Peak assignments following CID MS/MS of *m/z* 715.0316
