# Supplementary material for: Subcritical Water Hydrolysis of Peptides: Amino Acid Side-Chain Modifications
Source: J Am Soc Mass Spectrom. 2017 May 17;28(9):1775–86. doi: 10.1007/s13361-017-1676-1 (PMC5556142; doi:10.1007/s13361-017-1676-1)
Supplement: Supplementary file 3 — (PPTX 372 kb) [file 13361_2017_1676_MOESM3_ESM.pptx]

## Slide 1
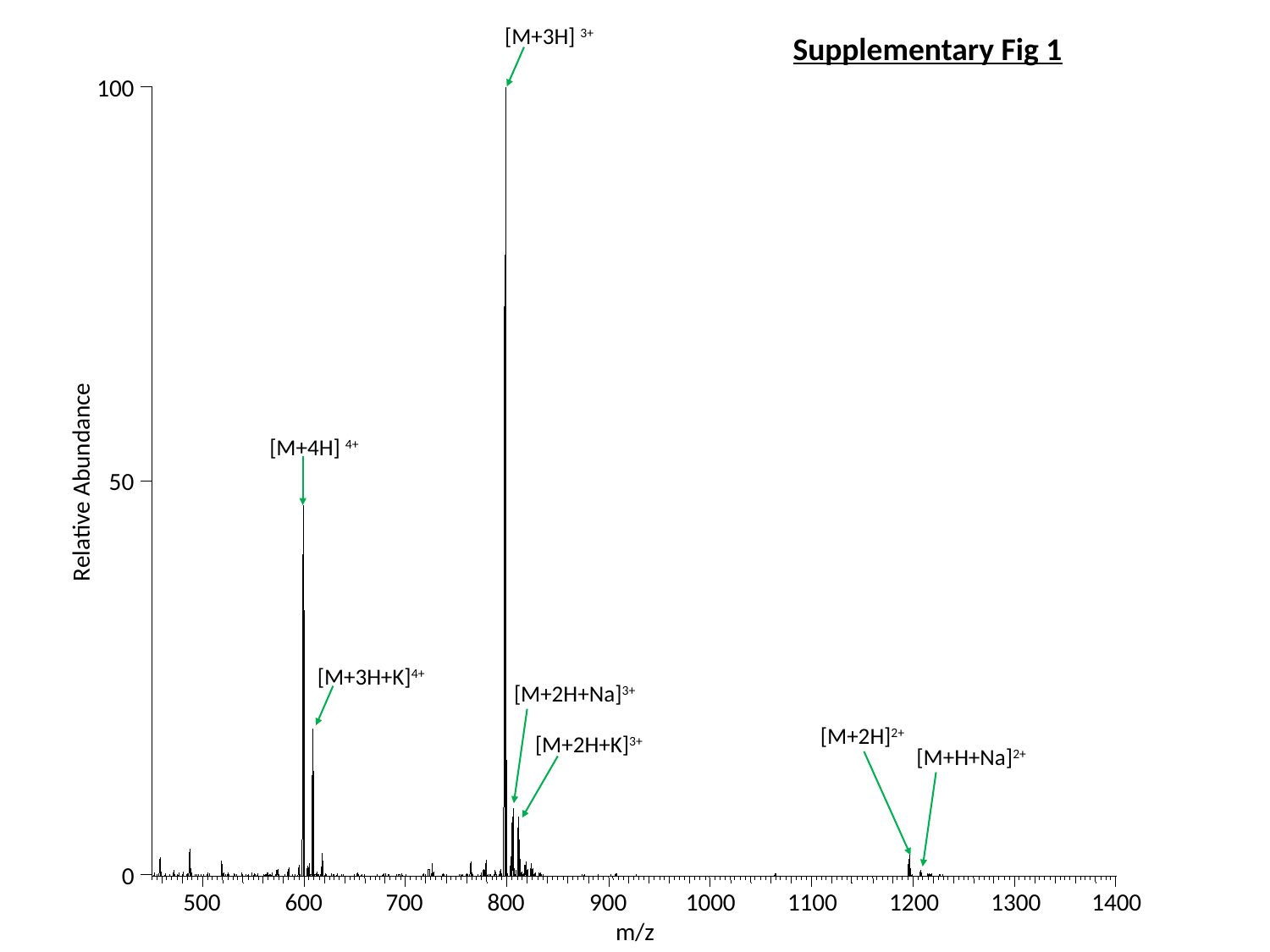

[M+3H] 3+
Supplementary Fig 1
100
[M+4H] 4+
50
Relative Abundance
[M+3H+K]4+
[M+2H+Na]3+
[M+2H]2+
[M+2H+K]3+
[M+H+Na]2+
0
500
600
700
800
900
1000
1100
1200
1300
1400
m/z

## Slide 2
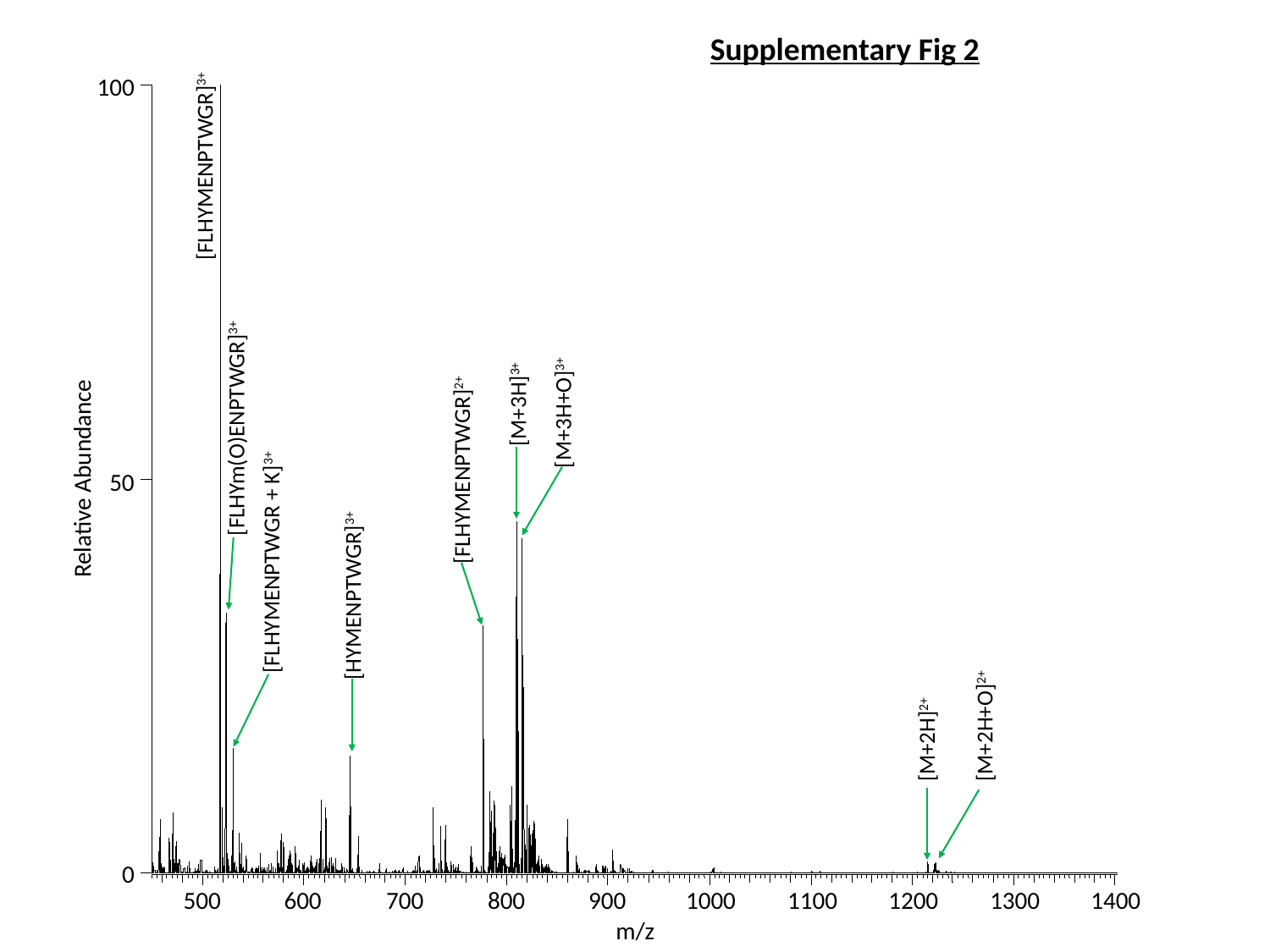

Supplementary Fig 2
100
[FLHYMENPTWGR]3+
[M+3H]3+
[M+3H+O]3+
[FLHYm(O)ENPTWGR]3+
[FLHYMENPTWGR]2+
Relative Abundance
50
[FLHYMENPTWGR + K]3+
[HYMENPTWGR]3+
0
500
600
700
800
900
1000
1100
1200
1300
1400
m/z
[M+2H+O]2+
[M+2H]2+

## Slide 3
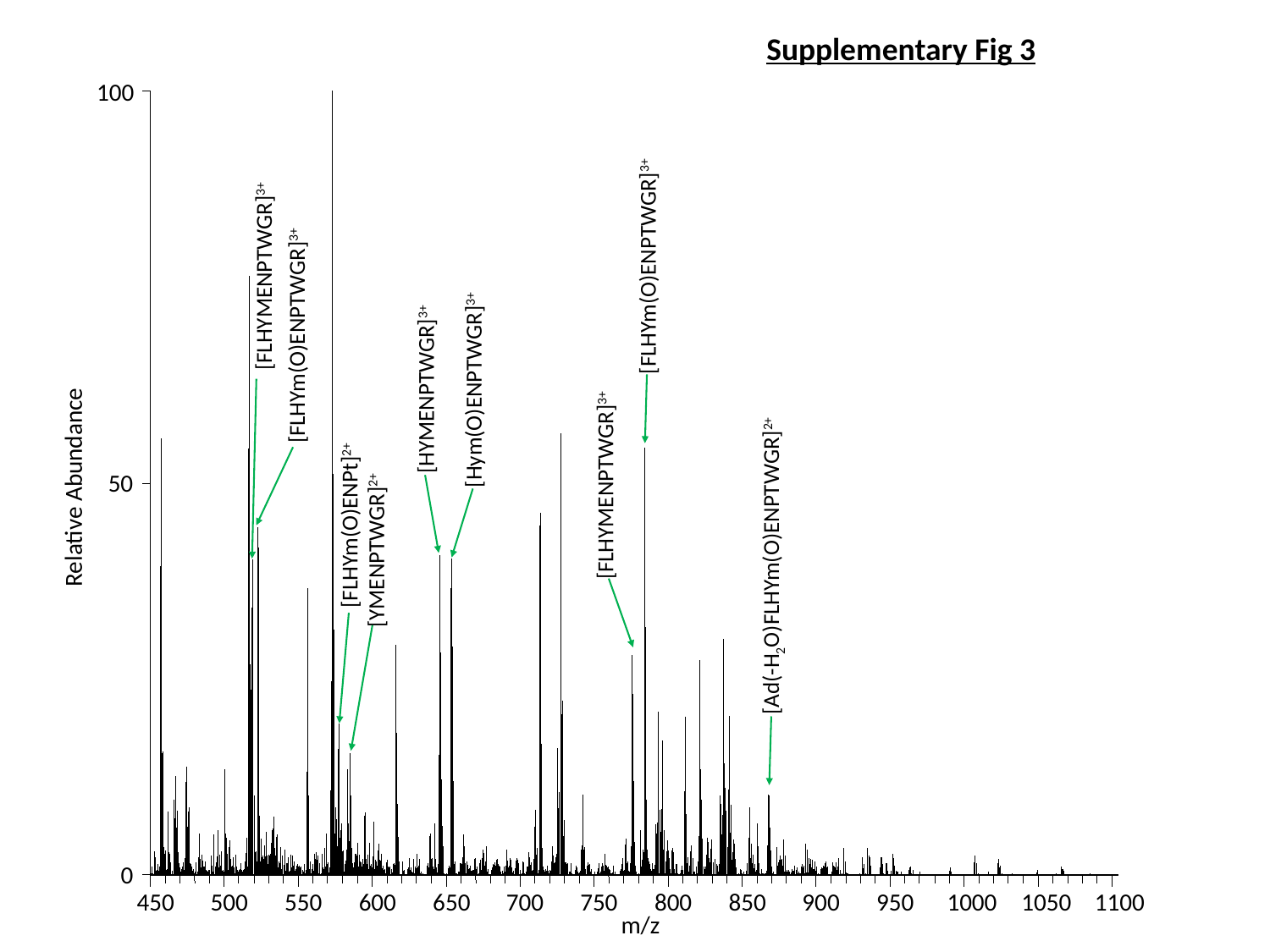

Supplementary Fig 3
100
[FLHYm(O)ENPTWGR]3+
[FLHYMENPTWGR]3+
[FLHYm(O)ENPTWGR]3+
[HYMENPTWGR]3+
[Hym(O)ENPTWGR]3+
[FLHYMENPTWGR]3+
50
Relative Abundance
[FLHYm(O)ENPt]2+
[YMENPTWGR]2+
[Ad(-H2O)FLHYm(O)ENPTWGR]2+
0
450
500
550
600
650
700
750
800
850
900
950
1000
1050
1100
m/z

## Slide 4
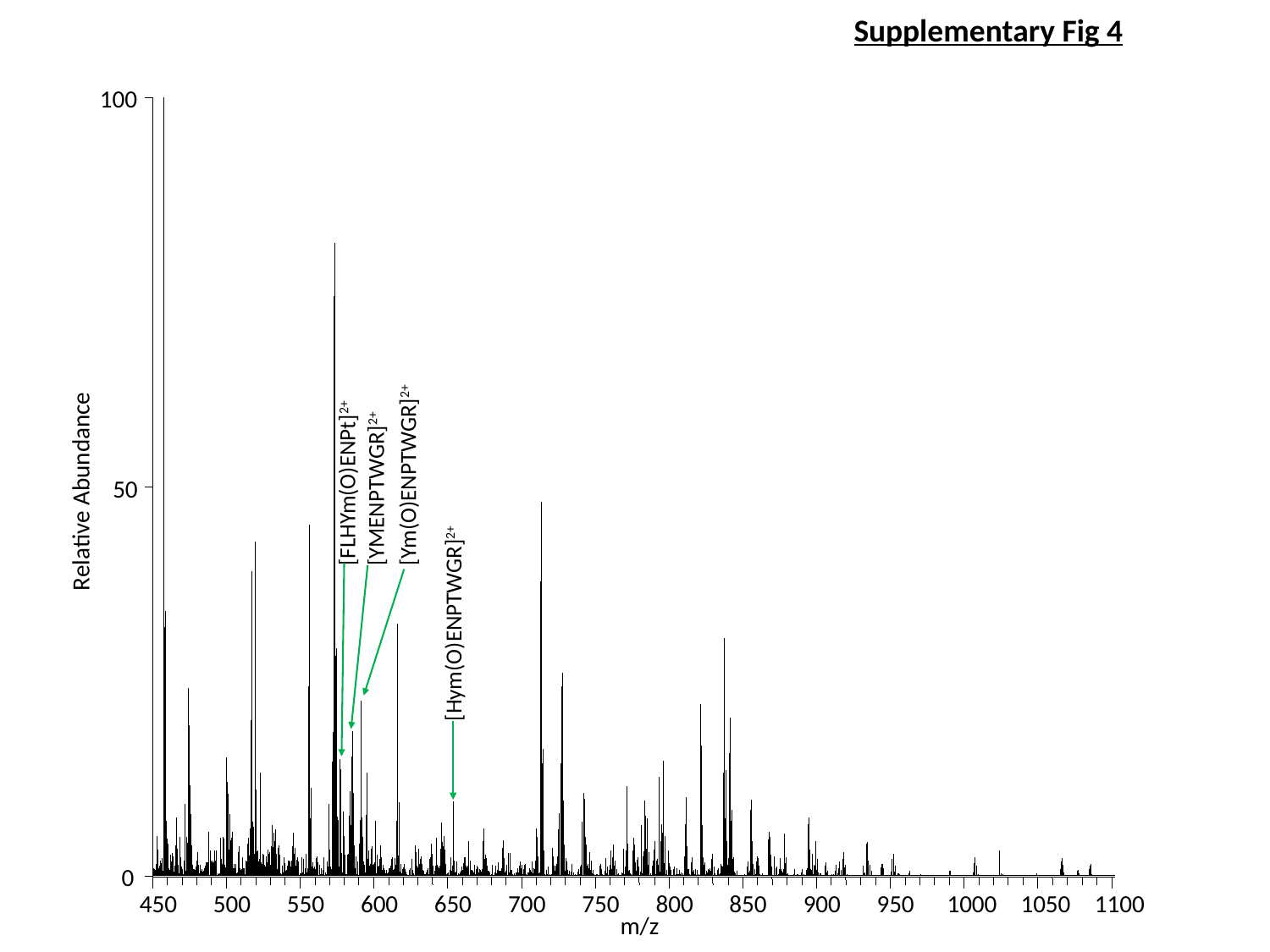

Supplementary Fig 4
100
[Ym(O)ENPTWGR]2+
[FLHYm(O)ENPt]2+
[YMENPTWGR]2+
50
Relative Abundance
[Hym(O)ENPTWGR]2+
0
450
500
550
600
650
700
750
800
850
900
950
1000
1050
1100
m/z

## Slide 5
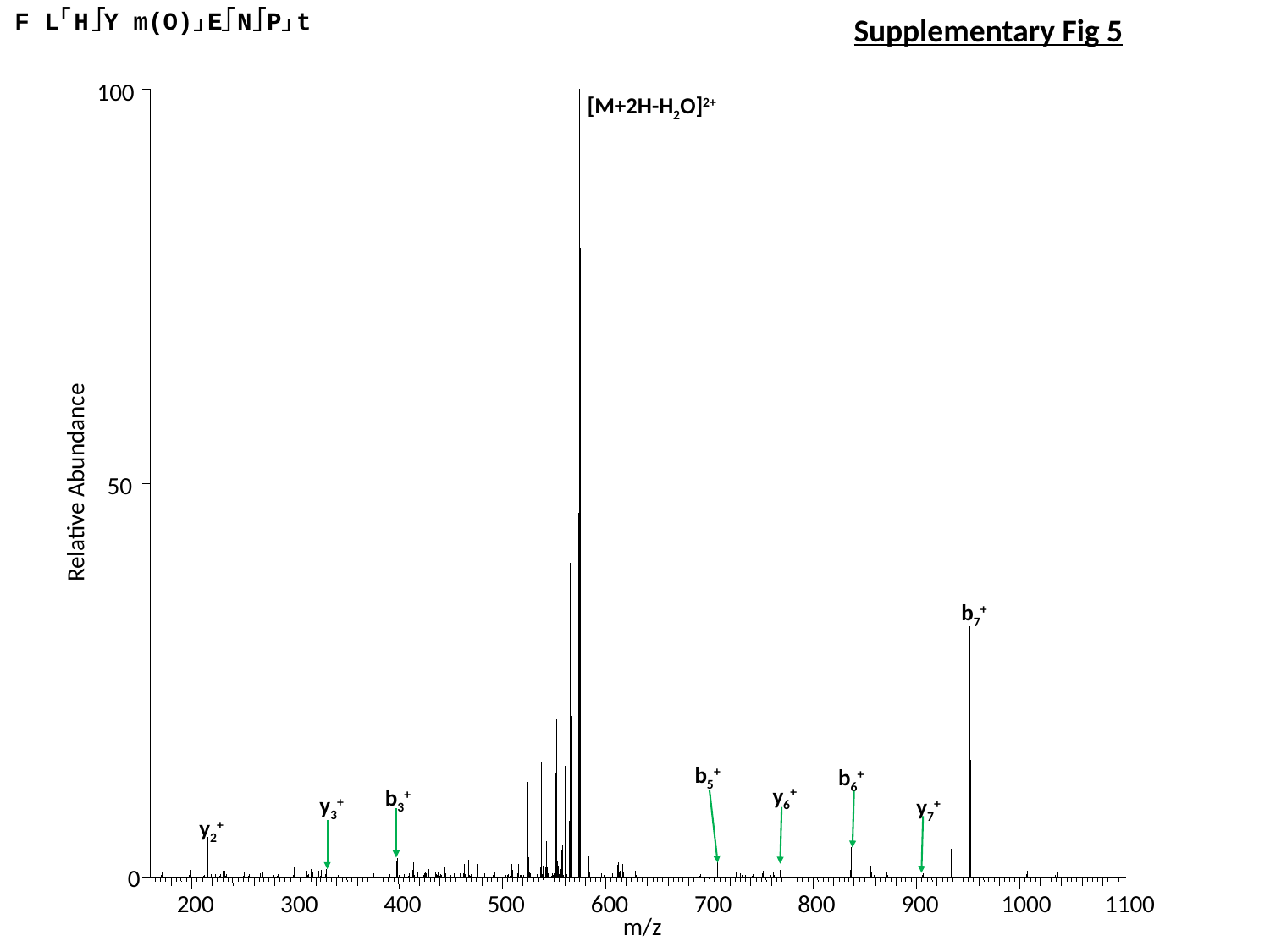

F L H Y m(O) E N P t
Supplementary Fig 5
100
[M+2H-H2O]2+
Relative Abundance
50
b7+
b5+
b6+
y6+
b3+
y3+
y7+
y2+
0
200
300
400
500
600
700
800
900
1000
1100
m/z

## Slide 6
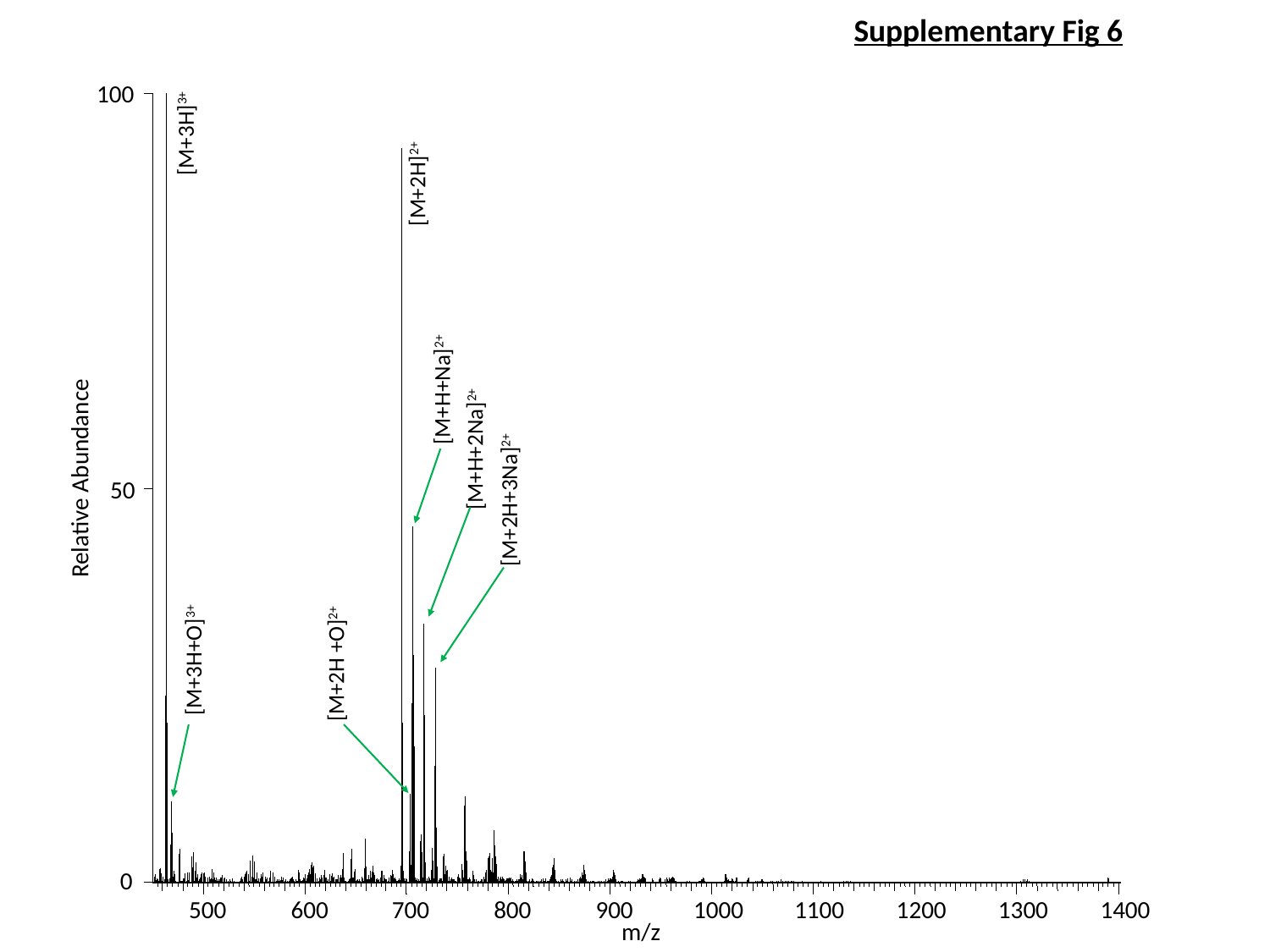

Supplementary Fig 6
100
[M+3H]3+
[M+2H]2+
[M+H+Na]2+
[M+H+2Na]2+
[M+2H+3Na]2+
Relative Abundance
50
[M+3H+O]3+
[M+2H +O]2+
0
500
600
700
800
900
1000
1100
1200
1300
1400
m/z

## Slide 7
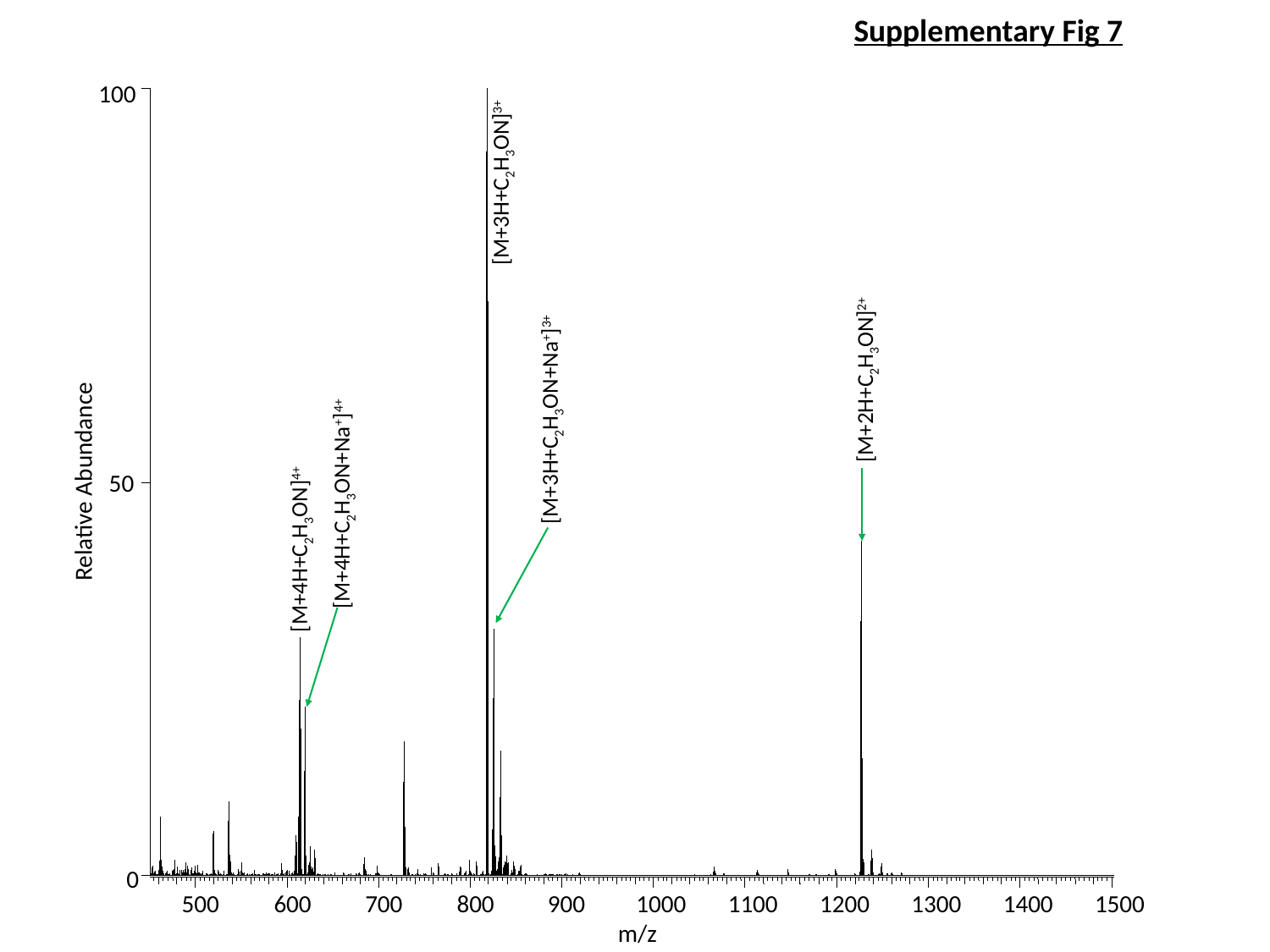

Supplementary Fig 7
100
[M+3H+C2H3ON]3+
[M+2H+C2H3ON]2+
[M+3H+C2H3ON+Na+]3+
Relative Abundance
50
[M+4H+C2H3ON+Na+]4+
[M+4H+C2H3ON]4+
0
500
600
700
800
900
1000
1100
1200
1300
1400
1500
m/z

## Slide 8
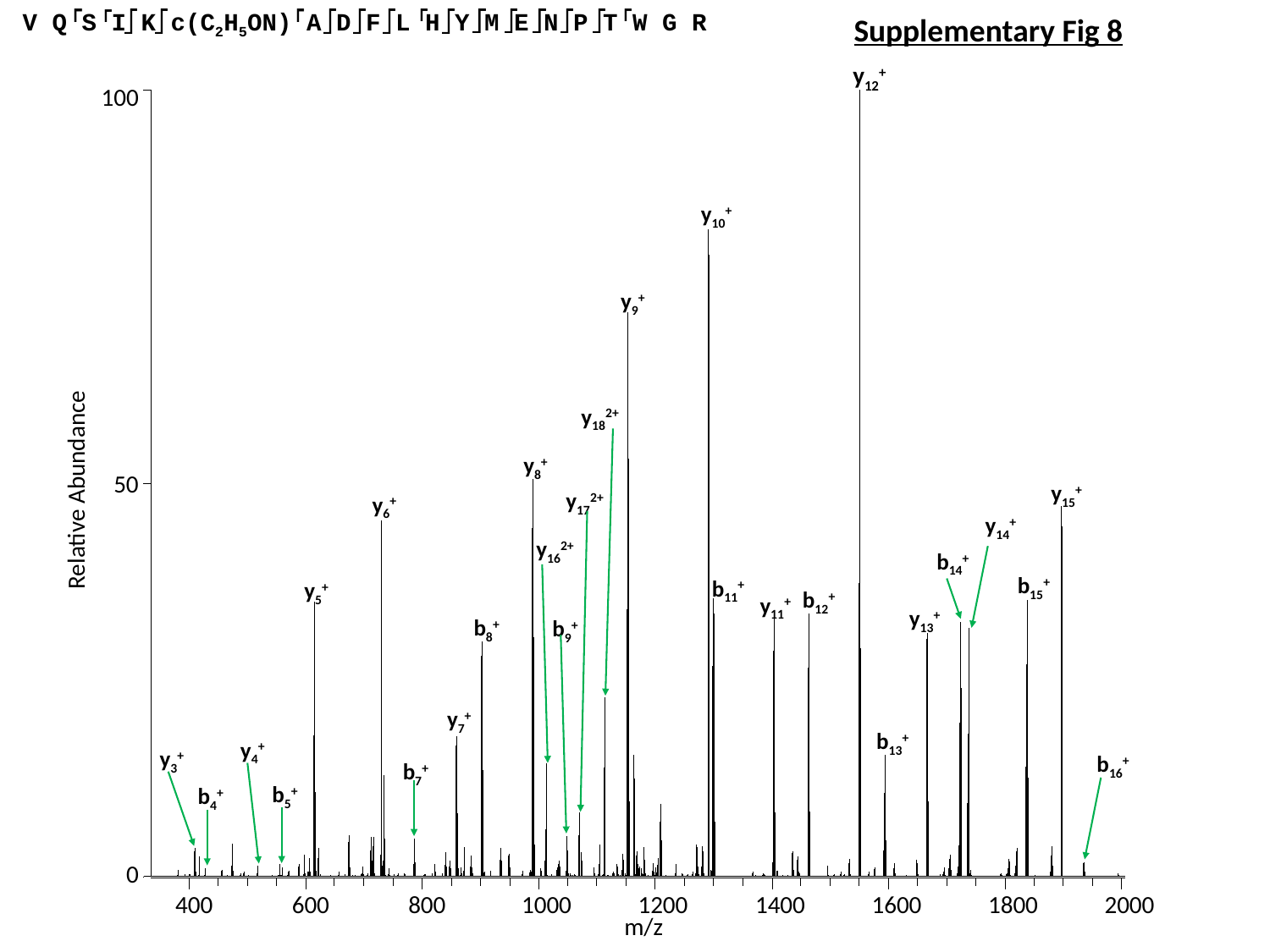

V Q S I K c(C2H5ON) A D F L H Y M E N P T W G R
Supplementary Fig 8
y12+
100
y10+
y9+
y182+
y8+
50
y15+
Relative Abundance
y172+
y6+
y14+
y162+
b14+
b15+
b11+
y5+
b12+
y11+
y13+
b8+
b9+
y7+
b13+
y4+
y3+
b16+
b7+
b5+
b4+
0
400
600
800
1000
1200
1400
1600
1800
2000
m/z

## Slide 9
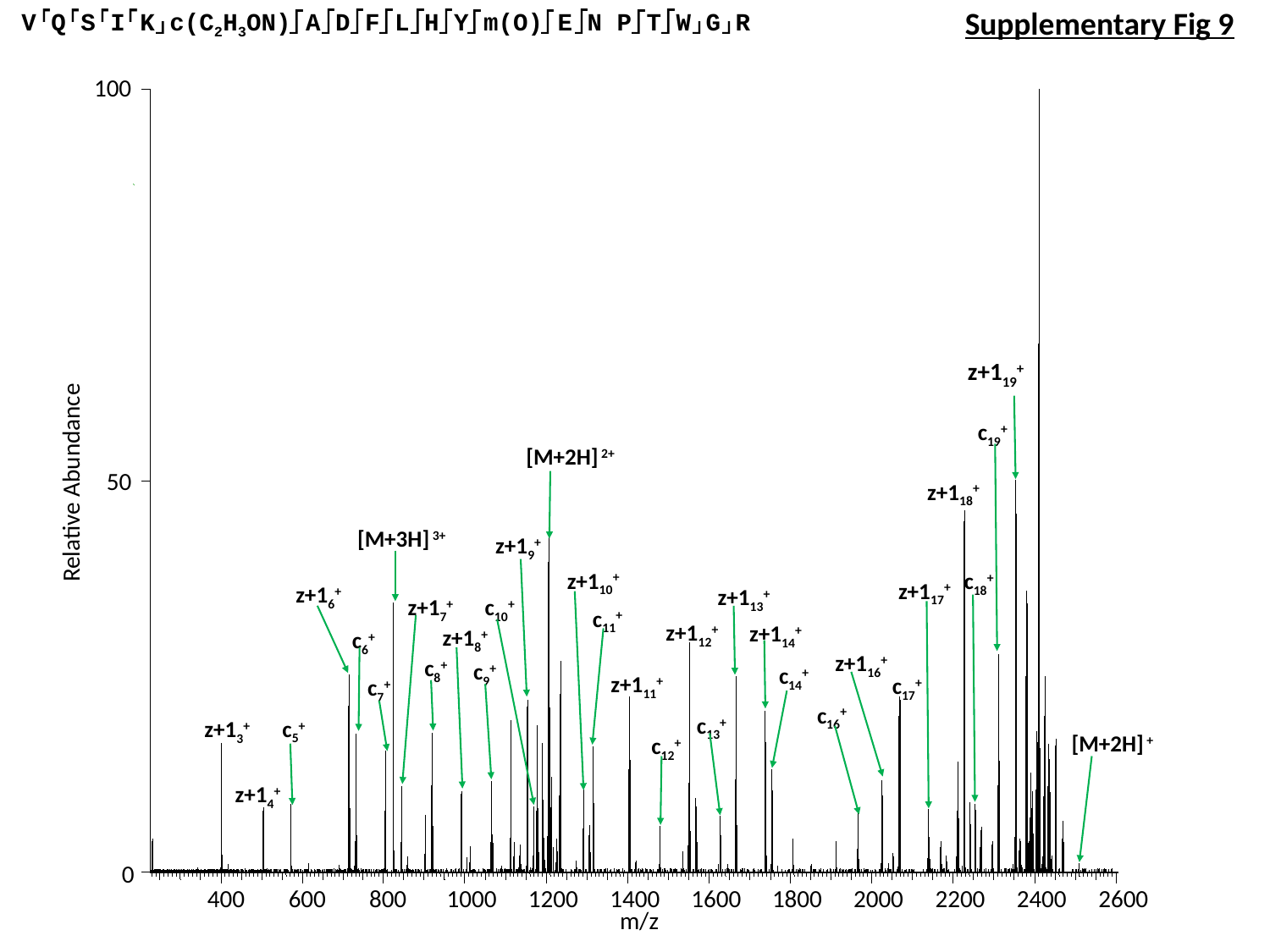

V Q S I K c(C2H3ON) A D F L H Y m(O) E N P T W G R
Supplementary Fig 9
100
z+119+
c19+
[M+2H] 2+
50
Relative Abundance
z+118+
[M+3H] 3+
z+19+
z+110+
c18+
z+117+
z+16+
z+113+
c10+
z+17+
c11+
z+112+
z+114+
z+18+
c6+
z+116+
c8+
c9+
c14+
z+111+
c17+
c7+
c16+
c13+
c5+
z+13+
[M+2H] +
c12+
z+14+
0
400
600
800
1000
1200
1400
1600
1800
2000
2200
2400
2600
m/z

## Slide 10
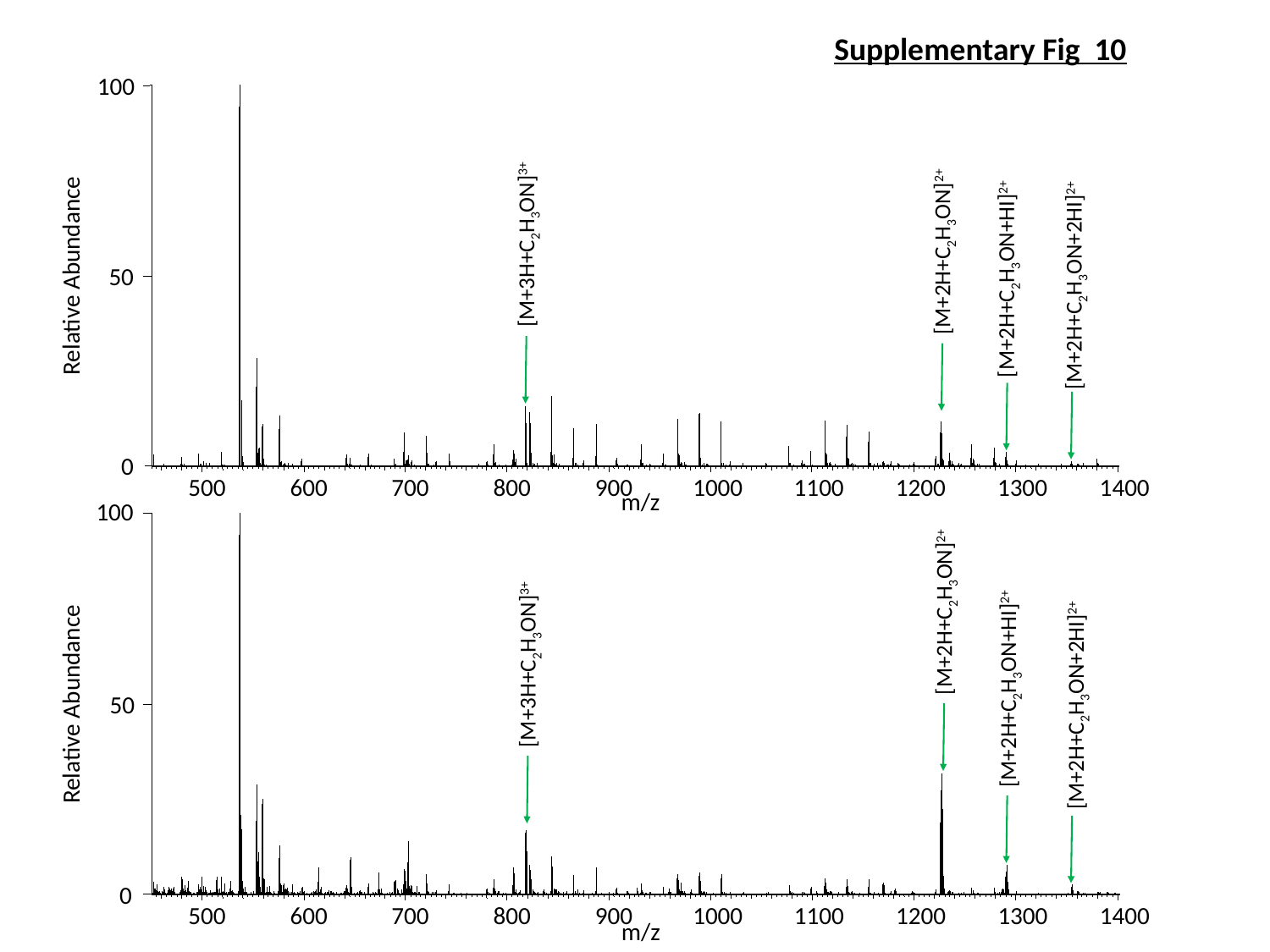

Supplementary Fig 10
100
[M+3H+C2H3ON]3+
[M+2H+C2H3ON]2+
[M+2H+C2H3ON+HI]2+
Relative Abundance
50
[M+2H+C2H3ON+2HI]2+
0
800
900
1000
1100
1200
1300
1400
500
600
700
m/z
100
[M+2H+C2H3ON]2+
[M+3H+C2H3ON]3+
[M+2H+C2H3ON+HI]2+
[M+2H+C2H3ON+2HI]2+
Relative Abundance
50
0
500
600
700
800
900
1000
1100
1200
1300
1400
m/z

## Slide 11
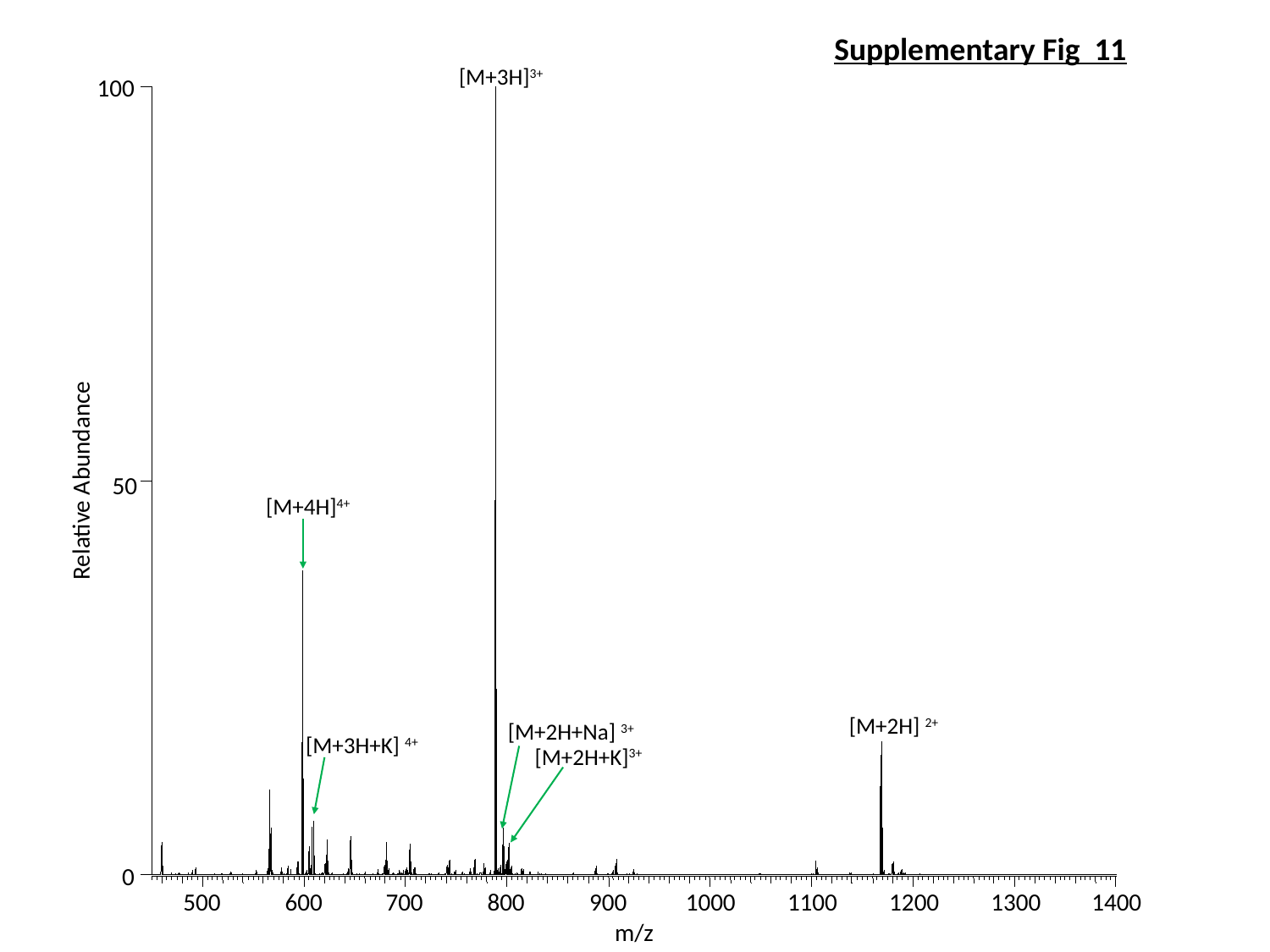

Supplementary Fig 11
[M+3H]3+
100
Relative Abundance
50
[M+4H]4+
[M+2H] 2+
[M+2H+Na] 3+
[M+3H+K] 4+
[M+2H+K]3+
0
500
600
700
800
900
1000
1100
1200
1300
1400
m/z

## Slide 12
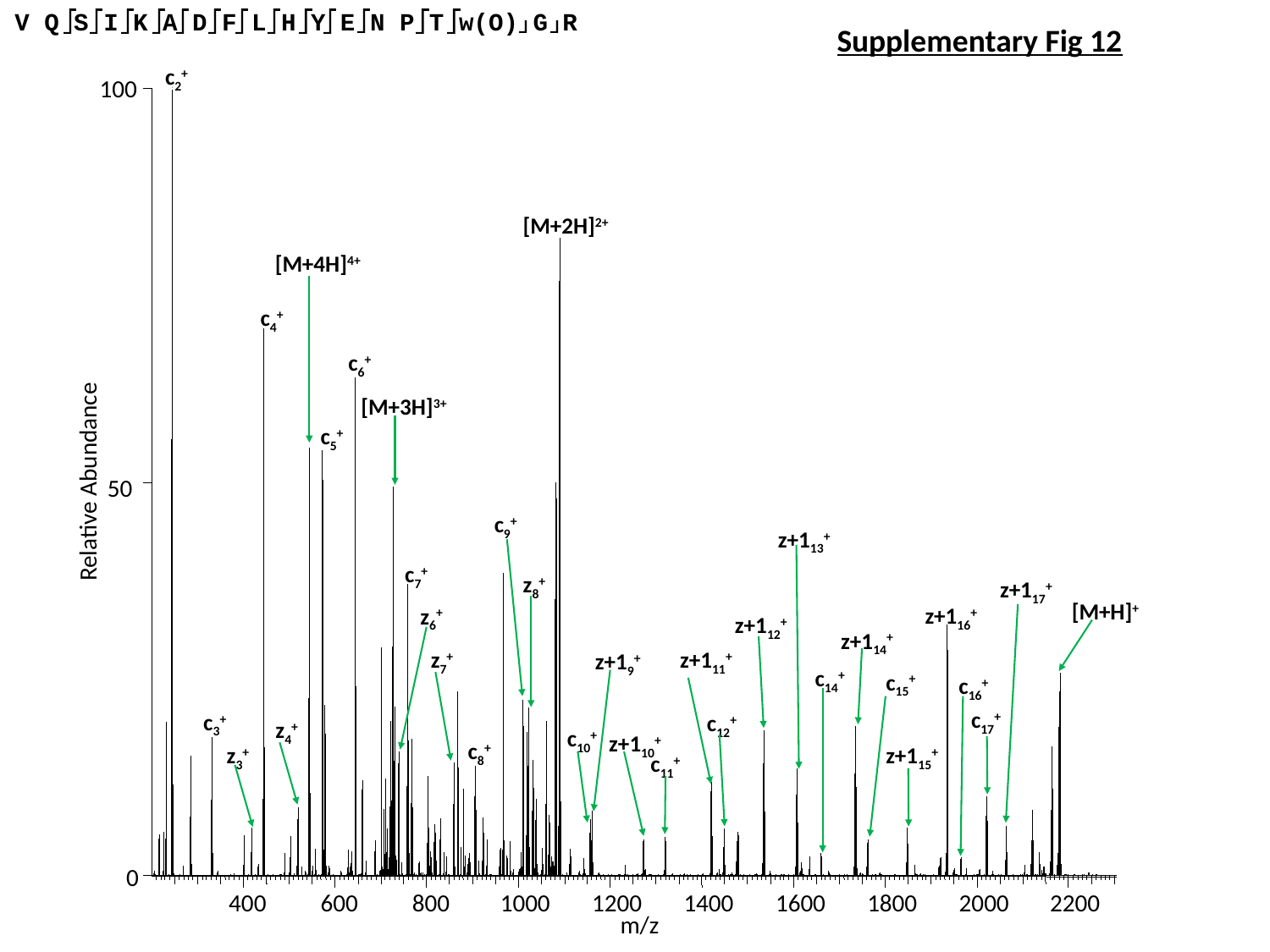

V Q S I K A D F L H Y E N P T w(O) G R
Supplementary Fig 12
c2+
100
[M+2H]2+
[M+4H]4+
c4+
c6+
[M+3H]3+
c5+
Relative Abundance
50
c9+
z+113+
c7+
z8+
z+117+
[M+H]+
z+116+
z6+
z+112+
z+114+
z+111+
z7+
z+19+
c14+
c15+
c16+
c17+
c3+
c12+
z4+
c10+
z+110+
c8+
z3+
z+115+
c11+
0
400
600
800
1000
1200
1400
1600
1800
2000
2200
m/z

## Slide 13
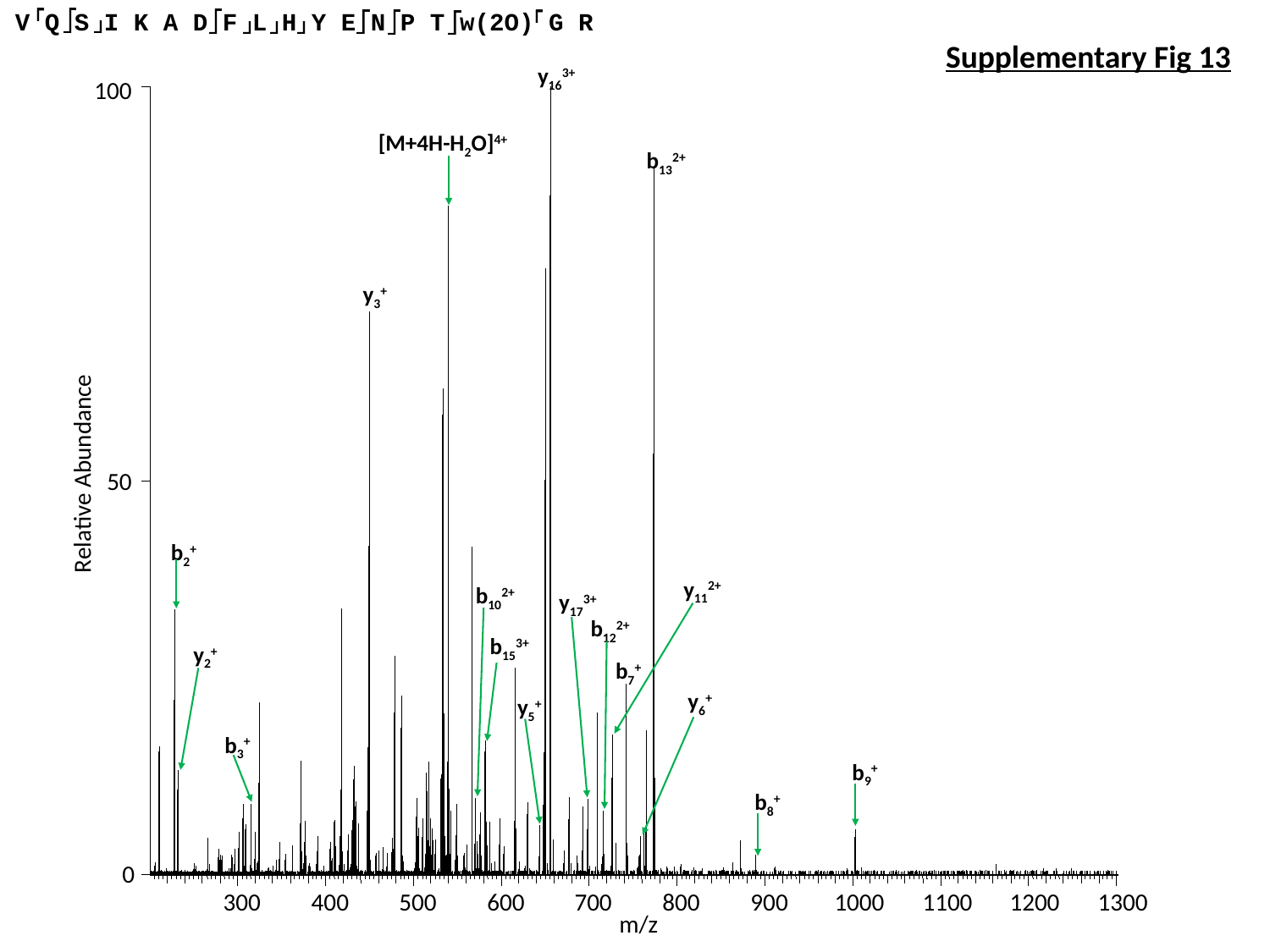

V Q S I K A D F L H Y E N P T w(2O) G R
Supplementary Fig 13
y163+
100
[M+4H-H2O]4+
b132+
y3+
Relative Abundance
50
b2+
y112+
b102+
y173+
b122+
b153+
y2+
b7+
y6+
y5+
b3+
b8+
0
300
400
500
600
700
800
900
1000
1100
1200
1300
m/z
b9+

## Slide 14
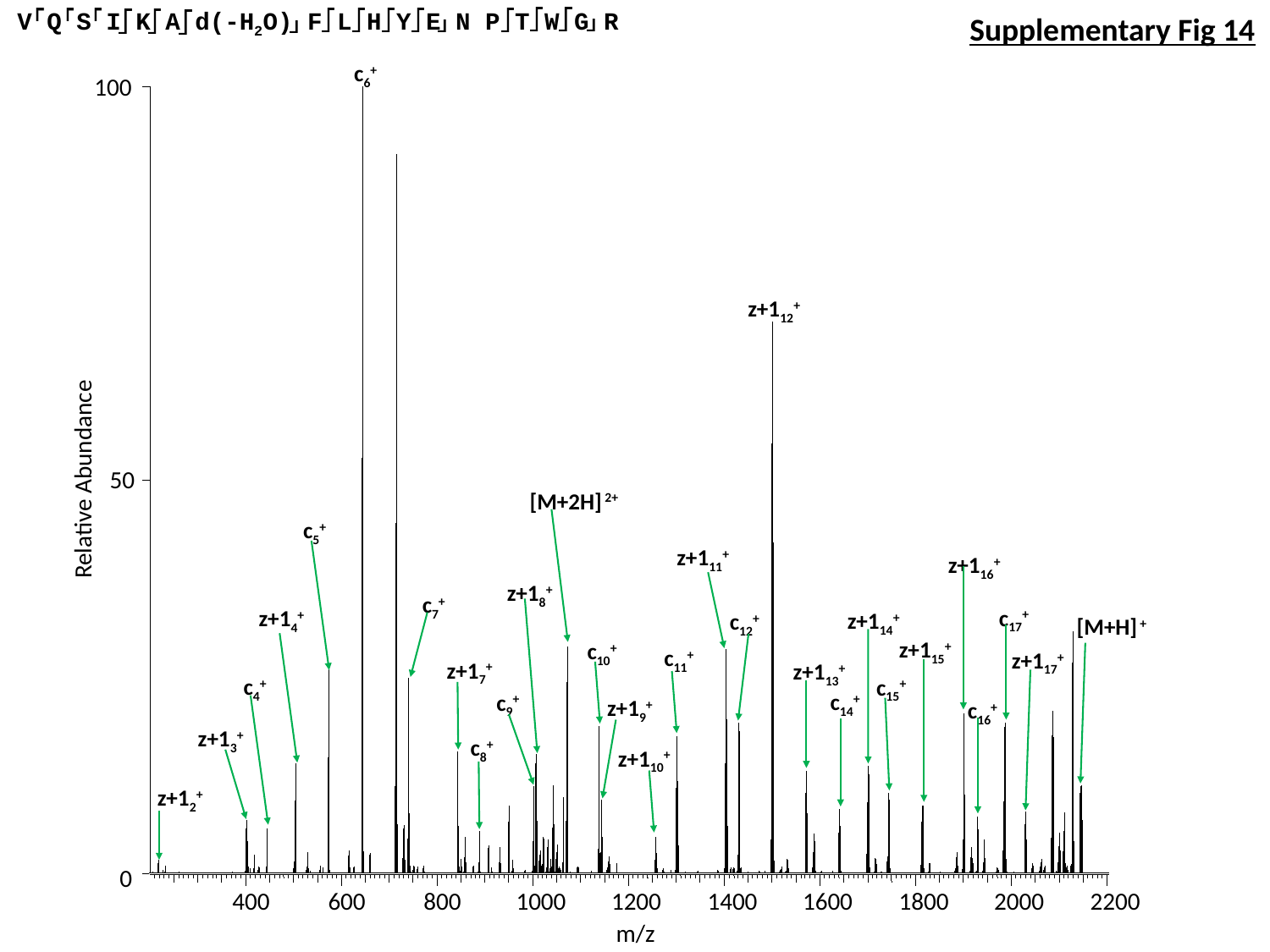

V Q S I K A d(-H2O) F L H Y E N P T W G R
Supplementary Fig 14
c6+
100
z+112+
50
Relative Abundance
[M+2H] 2+
c5+
z+111+
z+116+
z+18+
c7+
c17+
z+14+
z+114+
c12+
[M+H] +
z+115+
c10+
c11+
z+117+
z+17+
z+113+
c4+
c15+
c14+
c9+
z+19+
c16+
z+13+
c8+
z+110+
z+12+
0
400
600
800
1000
1200
1400
1600
1800
2000
2200
m/z
